# Linear Paired Electrolysis—Realising 200 % Current Efficiency for Stoichiometric Transformations—The Electrochemical Bromination of Alkenes

**DOI:** 10.1002/anie.202016413

**Published:** 2021-03-24

**Authors:** Julia Strehl, Marvin L. Abraham, Gerhard Hilt

**Affiliations:** ^1^ Institut für Chemie Universität Oldenburg Carl-von-Ossietzky-Strasse 9–11 26111 Oldenburg Germany

**Keywords:** 200 % cell, alkene, arene, bromination, linear paired electrolysis

## Abstract

The generation of bromine by oxidation of bromide anions at the anode and reduction of molecular oxygen at the cathode to hydrogen peroxide resulted in the overall formation of two molecules of Br_2_ (=four electron oxidation) by passing just two electrons through the solution. The bromine was used for the bromination of alkenes and thereby a linear paired electrolysis was attained which resulted in current efficencies of up to 200 %. Also, the diiodination of cyclohexene as well as the electrophilic aromatic bromination of an electron‐rich arene were realised both in 168 % current efficiencies.

## Graphical abstract

Bromination of Alkenes 2.0—can a stoichiometric chemical transformation exceed a 100 % yield? No! But there is a way to achieve an eletrochemical current efficiency of up to 200 % when oxygen is used as the sacrifical starting material. The cathodic reduction of oxygen to H_2_O_2_ led to the bromination of alkenes in a linear paired electrolysis in up to perfect 200 % current efficiency.

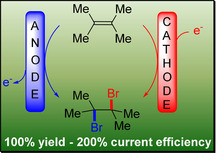

Electrochemical transformations are coupled processes where either an oxidation or a reduction represents the centre of interest. In most cases the electrochemical reaction at the counter electrode is of little concern (e.g. generation of hydrogen from protons).^[1]^ In contrast, in a paired electrolysis both electrochemical reactions are producing valuable products and various types of such transformations have been realised.^[2]^ For example, in a convergent paired electrolysis a single product (Nu−E) can be formed when a nucleophile (Nu^−^) is formed at the cathode and an electrophile (E^+^) at the anode and the reaction of these species in the bulk solution leads to the product. In this case, 100 % of the electrons supplied by the cathode and 100 % of the electrons absorbed at the anode are responsible for the production of the product and an overall current efficiency of 200 % is possible—at least in principle.

A more complicated case to realise is an oxidation, such as the conversion of a single substrate to a single product with 200 % current efficiency. In this case the anodic reaction as well as the cathodic reaction should generate the same product (Scheme 1). This paradoxon can only be solved by a sacrificial substance.

**Scheme 1:**
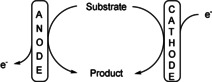
Linear paired electrolysis.

A seminal example for such a linear paired electrolysis with a theoretical 200 % current efficiency was published by Nonaka who investigated the formation of nitrones from hydroxylamines (Scheme 2).^[3]^

**Scheme 2:**
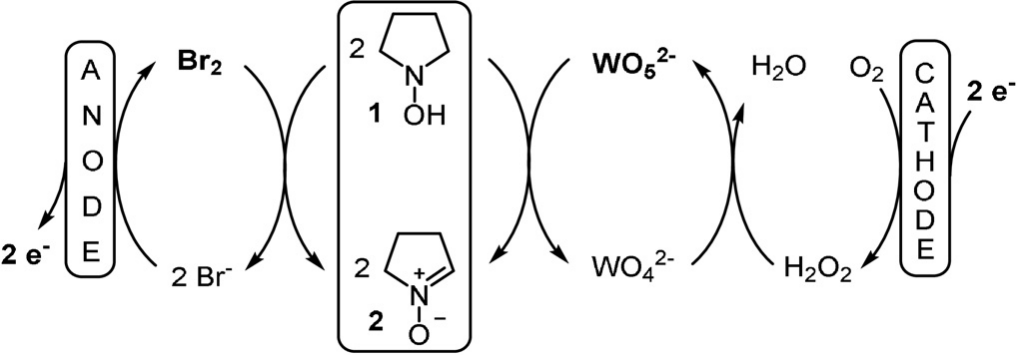
Linear paired electrolysis for the oxidation of hydroxylamines.

The oxidation of bromide ions to bromine at the anode accomplished the oxidation of the hydroxylamine **1** at the anode. Using molecular oxygen as sacrificial substance, the reduction at the cathode led to the formation of hydrogen peroxide. In combination with tungstenate, a peroxo‐tungstenate was proposed to accomplish the oxidation of the substrate **1** “via a reductive process”. The authors reported a current efficiency (ce) of up to 185 % for the formation of the desired nitrones **2**.^[4]^

Another example of a 200 %‐cell linear paired electrolysis, that can be found in the literature concerning organic transformations, was reported in a diploma thesis by Arns from the Steckhan group in 1998 (Scheme 3).^[5]^ The results for the oxidation of furane in up to 195 % current efficiency were never published elsewhere and represent the basis for this investigation (Scheme 3).

**Scheme 3:**
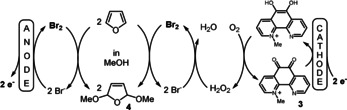
Linear paired electrolysis for the oxidation of furane.

As a catalyst for the reduction of molecular oxygen a *N*‐methylated 1,10‐phenanthroline‐5,6‐dione catalyst (**3**; PDMe) was utilised and in methanol solution furane was converted to the dimethoxy derivative **4** in good overall yield. However, when we adopted this reaction for the bromination of cyclohexene[^6^, ^7^] we recognised some inconsistencies. The outline of the desired reaction is shown in Scheme 4.

**Scheme 4:**
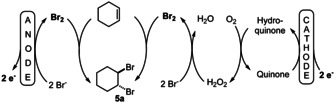
Quinone‐mediated linear paired electrolysis for the bromination of cyclohexene.

The current demand for high efficiencies in chemistry and the preservation of energy in general are of high interest. Therefore, the perspective of doubling the efficiency of a chemical reaction by an innovative technique, such as a linear paired electrolysis, was investigated.

A linear paired electrolysis is principally performed in an undivided cell design and passing 1.0 *F* through the solution would result in complete conversion of the cyclohexene giving a 200 % current efficiency. However, when the reaction is performed in a divided cell, the efficiency of the anodic as well as the cathodic reaction can be determined independently. Also, in a divided cell 2.0 *F* have to be passed through the solution to obtain theoretical 100 % conversion. Accordingly, the optimisation of the linear paired electrolysis was first conducted in a divided cell and later in an undivided cell to determine the efficiency of the coupled processes. In fact, the presence of quinones, such as **3** or anthraquinone, which was reported to facilitate the formation of H_2_O_2_,^[8]^ proved to be effective for the bromination of cyclohexene in the cathode compartment but the effect was marginal, so that the further reactions were conducted in the absence of these quinones (for details, see SI).

The oxidation of bromide to bromine in the anode compartment of a divided cell, where bromine equilibrates with the tribromide anion (Br_3_^−^),^[9]^ proved to be uncritical and therefore, we turned our attention to the cathode compartment to optimise the reduction of oxygen to H_2_O_2_ to produce bromine as well. The preliminary optimisation of the cathodic oxidation reaction was performed in a divided cell under oxygen atmosphere to verify good starting conditions for this part of the electrolysis. Subsequently, the extensive optimisation was realised in an undivided cell design and is summarised in Table 1 (for further results see SI). First, we tested several bromide sources (NaBr, KBr, and ***n*** **Bu_4_NBr**) and solvents (DMF, TFE, HFIP, and **CH_3_CN**) and their water content (0–20 equiv), as well as the reaction temperature (−12 to +20 °C). Afterwards, the influence of the electrode distance (1–3 cm), the electrode surface (3.5–6.0 cm^2^), the current (5–15 mA), and the stirring rate (150–750 rpm) were examined. The best results for the temperature dependence of the reaction were obtained at −5 °C, but for general convenience, we decided to perform the electrolysis at 0 °C with an ice bath and not at higher or lower temperatures (Table 1, entries 1–3). The product generation was mostly influenced by the surface areas of the electrodes (Table 1, entries 4–7), less by the electrode distance or the electrode material itself. Reducing the electrode surface of the platinum anode by about 50 % resulted in almost doubling of the current density and led to low overall performance while small variations of the current (Table 1, entries 8/9) had a comparable little effect. With respect to yield and current efficiency the amount of sulfuric acid was found to be optimal at 0.2 m. A factor that is quite relevant and is overlooked (or irrelevant) in many organic reactions is the stirring rate (the size of the stir bar^[10]^ was kept constant), which intensively affects the mass transport. An electrochemical short‐cut (oxidising bromide to bromine at the anode, transport to the cathode and reduction of bromine to bromide—or the oxidation of H_2_O_2_ at the anode) had to be avoided. Therefore, we checked the stirring rate as well and the best results were obtained when 250 rpm were applied. Slower stirring might cause a too slow mass transport of the cathodically generated H_2_O_2_ into the bulk solution where Br_2_ is formed. If this process happens near the cathode, reduction of Br_2_ to bromide can occur and a reduced current efficiency is observed. Faster stirring rates led to a diminished overall performance as well, probably by a faster mass transport of the electrogenerated species to the respective counter electrode (see SI).

**Table 1:** Results of the electrochemical bromination of cyclohexene in a linear paired electrolysis. 
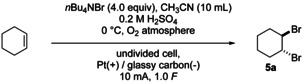

Entry	Variations from initial conditions	Yield^[a]^
1	none	75 % (ce: 150 %)
2	−5 °C	79 % (ce: 158 %)
3	15 °C	66 % (ce: 131 %)
4	electrode distance: 9 mm^[b]^	80 % (ce: 160 %)
5	glassy carbon cathode (1.50 cm^2^)^[c]^	81 % (ce: 162 %)
6	Pt anode (1.50 cm^2^)	24 % (ce: 48 %)
7	both electrodes (1.50 cm^2^)	35 % (ce: 70 %)
8	7.5 mA	71 % (ce: 142 %)
9	12 mA	81 % (ce: 162 %)
10	0.1 m H_2_SO_4_	65 % (ce: 130 %)
11	0.3 m H_2_SO_4_	67 % (ce: 134 %)
12	150 rotations/min	81 % (ce: 162 %)
**13**	**250 rotations/min**	**95 %^[d]^ (ce: 190 %)**

Unless otherwise stated, the reactions were performed in an undivided cell with a platinum anode (active surface area: 2.55 cm^2^) and a glassy carbon electrode (active surface area: 2.55 cm^2^) with an electrode distance of 11 mm on a 0.5 mmol scale. The reaction mixture was saturated with oxygen for 10 minutes at 0 °C prior to electrolysis. [a] The yield was determined by GC analysis of the crude reaction mixture using mesitylene as internal standard. [b] This change was kept for entries 5–13. [c] This change was kept for entries 6–13. [d] Isolated yield.

The scope of the linear paired electrolysis for the bromination of alkenes was investigated following the reaction outlined in Scheme 5.

**Scheme 5:**
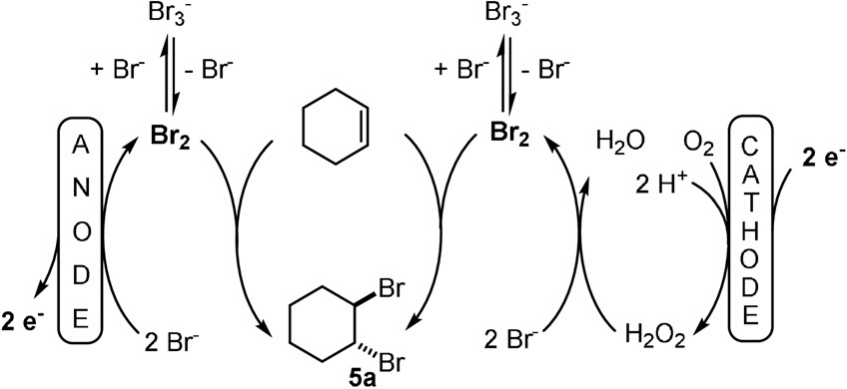
Linear paired electrolysis for the bromination of cyclohexene.

On a semi‐preparative scale of 0.5 mmol the electrolysis was conducted at 10 mA utilising a platinum anode (active surface area: 2.55 cm^2^), and a glassy carbon cathode (active surface area: 1.50 cm^2^; electrode distance 9 mm)^[11]^ to pass 1.0 *F* (per carbon−carbon double bond) through the solution.

The results of these electochemical bromination reactions are summarised in Scheme 6.

**Scheme 6:**
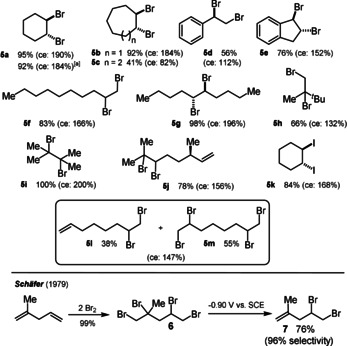
Linear paired electrolysis for the bromination of alkenes. [a] 2.5 mmol scale.

The linear paired electrolysis of cyclic alkenes (**5 a**–**5 c**) provided good to excellent results with the exception of cyclooctene for no obvious reason. Larger‐scale bromination was performed for **5 a** and resulted in a slightly diminished yield of 92 % (ce: 184 %) on a 2.5 mmol scale. While the bromination of styrene is performed in student laboratories worldwide within minutes and in almost quantitative yield, the linear paired electrochemical version, which takes around 80 minutes, led to unidentified side‐products and a low current efficiency of only 112 % for **5 d**. Terminal alkenes as well as 1,1‐disubstituted alkenes led to good to acceptable results as well (**5 f**/**5 h**). However, 5‐decene proved to be an excellent substrate. With 98 % isolated yield of **5 g** after passing 1.0 *F* electricity through the solution a current efficiency of 196 % was obtained. A perfect current efficiency of 200 % was obtained for product **5 i**, indicating that the more electron‐rich the alkene the faster the bromination occurs thereby avoiding the electrochemical short‐cut. Next, we turned our attention towards non‐conjugated dienes, such as β‐citronellene, and experienced that the dibrominated product **5 j**, isolated in 78 % yield (ce: 156 %) with a high chemoselectivity (>95 %) was formed as the main product. The high chemoselectivity for the bromination of a trisubstituted double bond over a monosubstituted double bond might be caused by the slow generation of the Br_2_ over 80 minutes electrolysis whereas the drop‐wise addition of Br_2_ in a chemical bromination leads to low chemoselectivities.

In comparison with the outstanding work performed by Schäfer more than 40 years ago (bottom of Scheme 6), electrochemical methods are now able to generate a selective dibromination of dienes, such as **5 j**, via the herein provided methodology or a chemoselective electrochemical debromination (by an electrochemical E1cB mechanism) to generate product **7** under potential‐controlled conditions.^[12]^ Also, a linear paired electrolysis for the diiodination of cyclohexene was investigated and the desired product **5 k** was isolated in 84 % yield after passing 1.0 *F* of electricity through the solution, which resulted in a current efficiency of 156 %. Not at all surprisingly, based on the relatively high oxidation potential of chloride anions, an analogous dichlorination of cyclohexene failed.

Surprisingly, in chemical control reactions, cyclohexene reacted with H_2_O_2_ in the presence of bromide ions (reaction (1) in Scheme 7), but no brominated product **5** was formed. When sulfuric acid was added to this solution (reaction (2) in Scheme 7), almost complete conversion towards **5 a** was observed in a short period of time. The hypothesis that persulfate (Caro's acid) was formed in situ and acts as oxidising agent to generate bromine was disproven because the same fast reaction towards **5 a** was observed when HBr (reaction (3) in Scheme 7) was added instead of sulfuric acid to the cyclohexene/H_2_O_2_ solution. Accordingly, under the reaction conditions of the electrolysis an acidic medium is useful to facilitate the formation of bromine from the electrogenerated H_2_O_2_ and further catalysts or additives are not needed.

**Scheme 7:**
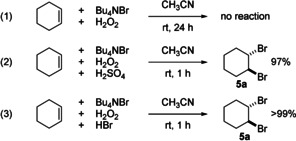
Chemical control experiments.

To identify functional group compatability, we then performed a tolerance test introduced by Glorius.^[13]^ Selected results are summarised in Table 2 (for further details see SI). Several functional groups were compatible with the linear electrolysis conditions for the bromination reaction of alkenes, such as alkynes, nitriles, esters, alkyl/aryl halides, and ethers. Some other functional groups led to moderate tolerance, such as nitrobenzene derivatives (based on the easy reduction of the nitro group) and electron‐rich arenes (see below). Epoxides were protonated by sulfuric acid and some side reactions occurred. For unknown reasons an aldehyde showed a considerable transformation to unidentified side‐products. Aniline and dodecylamine were protonated by the sulfuric acid and precipitated from the solution which looks like an incompatability. Nevertheless, when electron‐rich arenes were applied as additive in the compatability test, brominated side‐products could be identified by GCMS.

**Table 2:** Selected examples of a functional group tolerance test using cyclohexene as substrate.

Entry	Additive	Yield^[a]^ (additive)	Yield^[a]^ (product)
1	1‐dodecyne	96 %	84 %
2	octanenitrile	96 %	77 %
3	methylbenzoate	86 %	87 %
4	aniline	24 %	75 %
5	dodecylamine	7 %	77 %
6	1‐chlorooctane	91 %	79 %
7	*n*‐octanal	54 %	59 %
8	1,2‐epoxy‐*n*‐octane	66 %	80 %
9	valerophenone	79 %	84 %
10	dibutyl ether	83 %	72 %
11	1‐bromo‐1‐nitrobenzene	67 %	49 %
12	*N*,*N*‐dimethyl aniline	69 %	74 %

[a] The yield was determined by GC analysis of the crude reaction mixture using mesitylene as internal standard.

Accordingly, when 1,3‐dimethoxybenzene (**8**) was electrolysed under the conditions of the linear paired electrolysis the desired product **9** was obtained in 94 % yield utilising 1.2 *F*, which results in a current efficiency of 168 % (Scheme 8).^[14]^

**Scheme 8:**
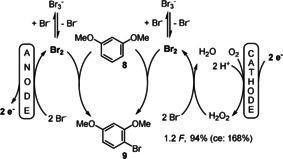
Linear paired electrolysis for the bromination of **8**.

In conclusion, we have optimised a linear paired electrolysis for the dibromination of alkenes in up to 100 % yield and a perfect current efficiency of 200 %. Several important reaction parameters were identified, optimised, and a functional group tolerance test identified other starting materials which can be applied in the linear paired bromination, such as electron‐rich arenes. In a preliminary reaction, the diiodination of cyclohexene was realised while the corresponding dichlorination was not. Nevertheless, in light of the current interest in electrochemical transformation, the increase of current efficiency for stoichiometric processes could be demonstrated.

## Conflict of interest

The authors declare no conflict of interest.

## Supporting information

As a service to our authors and readers, this journal provides supporting information supplied by the authors. Such materials are peer reviewed and may be re‐organized for online delivery, but are not copy‐edited or typeset. Technical support issues arising from supporting information (other than missing files) should be addressed to the authors.

Supplementary
