# Supplementary material for: Linear Paired Electrolysis—Realising 200 % Current Efficiency for Stoichiometric Transformations—The Electrochemical Bromination of Alkenes
Source: Angew Chem Int Ed Engl. 2021 Mar 24;60(18):9996–10000. doi: 10.1002/anie.202016413 (PMC8251945; doi:10.1002/anie.202016413)
Supplement: Supplementary file 1 — Supplementary [file ANIE-60-9996-s001.pdf]

Angewandte Chemie
Eine Zeitschrift der Gesellschaft Deutscher Chemiker

Supporting Information

Linear Paired Electrolysis—Realising 200 % Current Efficiency for Stoichiometric Transformations—The Electrochemical Bromination of Alkenes

*Julia Strehl, Marvin L. Abraham, and Gerhard Hilt**

anie_202016413_sm_miscellaneous_information.pdf

Supporting Information

Table of content

1. General Information S3

2. Electrosynthesis S5

   2.1. Preliminary Tests S5

   2.2. Optimization of the reaction conditions S7

   2.3. General procedure (GPA) for the electrochemical conversions S9

   2.4. Synthesized brominated products S10

   2.5. Robustness Screen S16

3. Mechanistic Investigations S18

   3.1. Control Experiments S18

   3.2. Cyclic voltammograms S19

4. NMR spectra of all synthesized compounds S22

5. References S36

1. General Information

All solvents were commercially available and have been distilled under reduced pressure prior to use. Solvents were dried over 3 Å molecular sieves.

All chemicals or reagents were purchased from commercial suppliers without further purification, if not otherwise stated, or were prepared according to known literature procedures. If water or air sensitive compounds have been used, the experiments were carried out in heat gun dried glassware using conventional SCHLENK techniques under nitrogen atmosphere. Electrochemical reactions were carried out using an AIM-TTI Instruments MX100T power supply. These reactions were performed in an undivided cell (Figure S1), equipped with a stirring bar, a platinum anode (1.70 · 3.50 cm^2, active surface: 2.55 cm^2) and a glassy carbon cathode (1.00 · 3.50 cm^2, active surface: 1.50 cm^2) (distance between the electrodes: 0.9 cm). All known compounds were characterized by ^{1}H and ^{13}C NMR. All unknown compounds were identified by ^{1}H NMR, ^{13}C NMR, IR and HRMS.

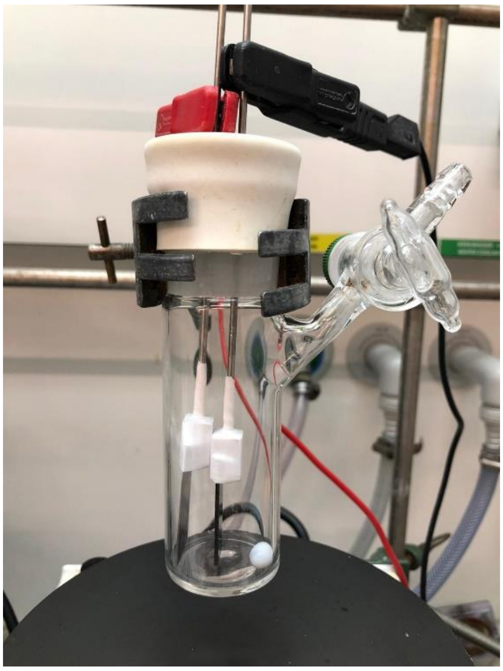

Figure S1: Undivided electrolysis cell, equipped with a platinum anode and a glassy carbon cathode.

NMR spectroscopy: NMR spectra were recorded either on a Bruker Avance 300 (300 MHz), on a Bruker Avance III (500 MHz) or on a Bruker Avance DRX (500 MHz). Chemical shifts are reported in parts per million (ppm). The spectra are referenced to the residual solvent peak of $CDCl_3$. In the ^{1}H NMR spectra this corresponds with the singlet of the solvent signal of $CDCl_3$ at δ = 7.26 ppm. The ^{13}C NMR spectra were referenced to the central line of the triplet of $CDCl_3$ at δ = 77.16 ppm.[1] The stated form of the signal describes the appearance of the signal and not the theoretically expected form.

IR Spectroscopy: The IR spectra were obtained with a Shimadzu IRSpirit with a QATR-S cell. The wave numbers λ^{-1} are quoted in reciprocal centimeters (cm^{-1}).

Chromatography: Flash chromatography was carried out using Machery-Nagel silica gel 60 (0.040-0.063 mm). Thin layer chromatography was carried out on Merck TLC plates coated with silica gel 60 F_{254} with fluorescence indicator. For the detection of the signals ultraviolet light (λ = 254 nm) or GC analysis were used or heating after the plate has been dipped into a $KMnO_4$-solution.

MS/HRMS: MS and HRMS spectra of products were obtained with a Waters Q-TOF Premier (ESI, pos. mode or APCI) or Thermo Scientific DFS (EI) spectrometers.

Cyclic voltammetry: Cyclic voltammetry measurements were carried out on a BAS C3 cell stand and a BAS 100 electrochemical analyzer using a glassy carbon disk working electrode (2.0 mm diameter) and platinum wire counter electrode (0.5 mm diameter). Potentials were referred to a saturated Ag/AgCl (3 M NaCl) reference electrode. Before each experiment, the solutions were purged with oxygen.

2. Electrosynthesis

2.1. Preliminary Tests

Table 1: Tests with PDMe (**3**) as catalyst.

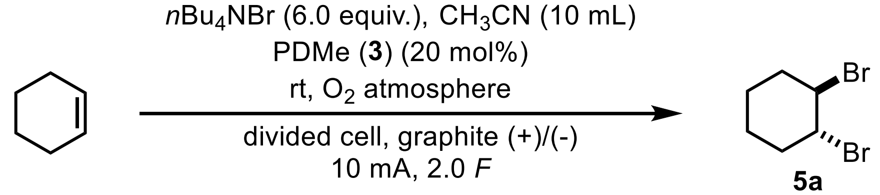

5a

Entry	Variation from the initial condition[a]	Yield[b]
1	None	traces
2	15 °C	3%
3	0 °C	5%
4	DMF[c]	3%
5	TFE[c]	-
6	HFIP[c]	traces
7	5 mA[c]	traces
8	20 mA[c]	traces
9	Without catalyst[c]	5%

PDMe (**3**) = *N*-methyl-1,10-phenanthroline-5,6-dione triflate. [a] The reactions were performed in a divided cell with graphite electrodes (active surface: 2.55 cm^2), with a stirring rate of 450 rpm and on a 0.50 mmol scale. The reaction mixture was saturated with oxygen for 10 minutes at 0 °C prior to electrolysis. [b] The yield was determined by GC analysis of the crude reaction mixture using mesitylene as internal standard. [c] The reaction was performed at 0 °C.

Table 2: Tests with anthraquinone as catalyst.

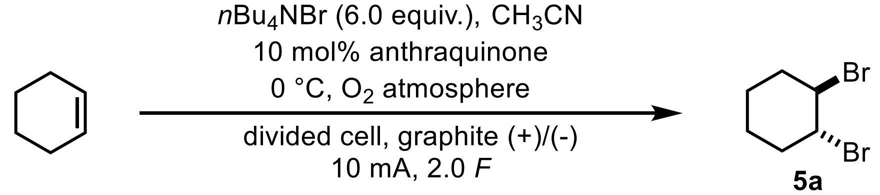

5a

Entry	Variation from the initial conditions[a]	Yield[b]
1	pH = 1, H_2SO_4 (0.2 M)	70%
2	pH = 2, phosphate buffer	0%
3	pH = 4, CH_3COOH	0%
4	pH = 7, phosphate buffer	0%
5	pH = 12, phosphate buffer	4%
6	pH = 14, KOH (aqueous solution)	0%

[a] The reactions were performed in a divided cell with graphite electrodes (active surface: 2.55 cm^2), with a stirring rate of 450 rpm and on a 0.50 mmol scale. The reaction mixture was saturated with oxygen for 10 minutes at 0 °C prior to electrolysis. [b] The yield was determined by GC analysis of the crude reaction mixture using mesitylene as internal standard.

Table 3: Optimization of the electrode material using anthraquinone as catalyst.

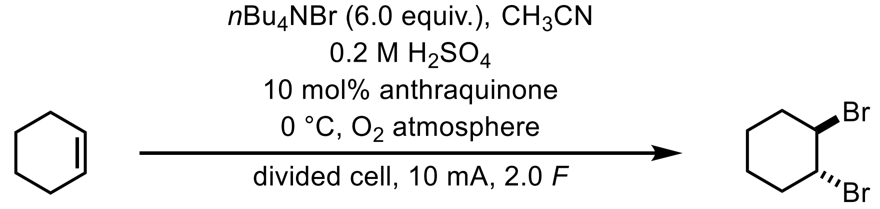

Entry	Electrode material	Yield (anode) [%][a]	Yield (cathode) [a]
1	graphite	83	53%
2	platinum	91	15%
3	nickel	traces	15%
4	copper	traces	22%
5	glassy carbon	11[b]	75%

The reactions were performed in an divided cell with a stirring rate of 450 rpm and a on a 0.5 mmol scale. The reaction mixture was saturated with oxygen for 10 minutes at 0 °C prior to electrolysis. [a] The yield was determinded by GC analysis of the crude reaction mixture using mesitylene as internal standard.

[b] No cyclohexene was added into the anodic compartment of the cell. This result shows, that the starting material or the product diffuse to some extent through the P4 frit. Because of this, the further optimization was performed in an undivided cell.

2.2. Optimization of the reaction conditions

Table 4: Optimization of the reaction conditions

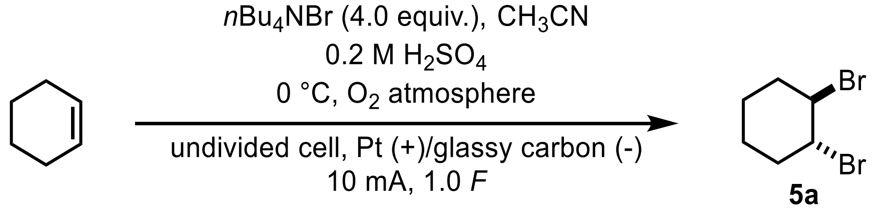

Entry	Variation from the initial conditions[a]	Yield of 5a[b] (CE)
1	none	75% (151%)
2	10 mol% anthraquinone	84% (168%)
	**Solvent**	
3	CH_2Cl_2	74% (148%)
4	DMF	51% (101%)
	**Amount of H_2O**	
5	anhydrous CH_3CN	54% (107%)
6	4.6 equiv. H_2O	70% (140%)
7	10 equiv. H_2O[c]	75% (150%)
8	15 equiv. H_2O	71% (141%)
9	20 equiv. H_2O	62% (124%)
	**Temperature**	
10	-12 °C	65% (131%)
11	-5 °C	79% (158%)
12	15 °C	66% (131%)
13	20 °C	56% (112%)
	**Electrode distance**	
14	9 mm[d]	80% (160%)
15	6 mm	80% (160%)
	**Electrode size**	
16	Active Surface (glassy carbon cathode) = 1.50 cm^2 [e]	81% (162%)
17	Active Surface (Pt anode) = 1.50 cm^2	24% (48%)
18	Active Surface (Pt anode and glassy carbon cathode) = 1.50 cm^2	35% (70%)
	**Amount of conc. H_2SO_4**	
19	0.1 M	65% (130%)
20	0.3 M	67% (135%)
	**Stirring rate**	
21	150 rpm	81% (162%)

22	250 rpm[f]	95% (186%)
23	450 rpm	81% (162%)
24	750 rpm	71% (142%)
	**Current**	
25	7.5 mA	71% (142%)
26	12 mA	81% (162%)
27	15 mA	73% (146%)
	**Equivalents of *n*Bu_4NBr**	
28	2 equiv.	68% (136%)
29	3 equiv.	80% (160%)
30	5 equiv.	79% (158%)
	**Bromide salt**	
31	NH_4Br	64% (128%)
32	NaBr	29% (57%)
33	KBr	10% (20%)
34	LiBr	6% (12%)
	**Reaction volume**	
35	5 mL	74% (148%)
36	7.5 mL	78% (156%)
37	12.5 mL	93% (186%)
38	15 mL	88% (176%)

CE = current efficiency. [a] The reactions were performed in an undivided cell with a platinum anode (active surface: 2.55 cm^2) and a glassy carbon electrode (active surface: 2.55 cm^2) and an electrode distance of 11 mm on a 0.5 mmol scale. The reaction mixture was saturated with oxygen for 10 minutes at 0 °C prior to electrolysis. [b] The yield was determinded by GC analysis of the crude reaction mixture using mesitylene as internal standard. [c] This change was kept for entries 10-38. [d] This change was kept for entires 16-38. [e] This change was kept for entries 19-38. [f] This change was kept for entries 25-38.

2.3. General procedure (GPA) for the electrochemical conversions

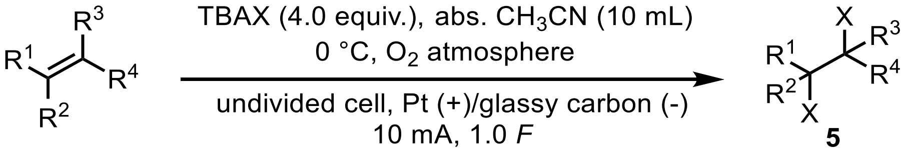

First, the tetrabutylammonium halide (TBAX) (2.00 mmol, 4.0 equiv.) was weighed into an undivided cell and dissolved in anhydrous acetonitrile (10 mL). Afterwards, water (0.09 mL, 90 mg, 5.0 mmol, 10 equiv.), conc. sulfuric acid (0.11 mL, 196 mg, 2.00 mmol) and the corresponding alkene (0.50 mmol, 1.0 equiv.) were added and the reaction mixture was cooled to 0 °C with an ice bath. At this temperature, the solution was saturated with oxygen by bubbling the gas through the solution for 10 minutes. Afterwards, the reaction mixture was electrolyzed under constant current (10 mA, 1.0 *F*, Pt cathode, glassy carbon anode) at 0 °C and with an oxygen atmosphere. The reaction mixture was diluted with saturated aqueous $Na_2S_2O_3$ solution (40 mL) and extracted with *n*-pentane (3 × 20 mL). The combined organic layers were dried ($MgSO_4$) and filtered. The residue was either submitted to column chromatography (SiO_2) or all volatile compounds were removed under reduced pressure to furnish the respective haloalkanes **5**.

2.4. Synthesized brominated products

1,2-Dibromocyclohexane (5a):

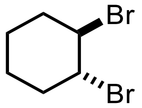

According to GPA, cyclohexene (41 mg, 0.50 mmol, 1.0 equiv.) and tetrabutylammonium bromide (645 mg, 2.00 mmol, 4.0 equiv.) were converted to furnish product **5a** (114 mg, 0.475 mmol, 95%, 190% CE) as colourless oil after evaporation of the solvent.

^{1}H NMR (300 MHz, $CDCl_3$): δ = 4.54-4.34 (m, 2 H), 2.54-2.34 (m, 2 H), 1.97-1.71 (m, 4 H), 1.62-1.40 (m, 2 H) ppm. **^{13}C NMR** (125 MHz, $CDCl_3$): δ = 55.3 (2 CH), 32.2 (2 CH_2), 22.5 (2 CH_2) ppm. The spectroscopic values are in accordance with literature values.[2]

1,2-Dibromoheptane (5b):

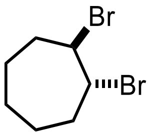

According to GPA, cycloheptane (48 mg, 0.50 mmol, 1.0 equiv.) and tetrabutylammonium bromide (645 mg, 2.00 mmol, 4.0 equiv.) were converted to furnish product **5b** (118 mg, 0.460 mmol, 92%, 184% CE) as colourless oil after column chromatography (SiO_2, *n*-pentane).

^{1}H NMR (300 MHz, $CDCl_3$): δ = 4.72-4.60 (m, 2 H), 2.43-2.27 (m, 2 H), 2.15-2.00 (m, 2 H), 1.96-1.77 (m, 2 H), 1.73-1.57 (m, 2 H) ppm. **^{13}C NMR** (125 MHz, $CDCl_3$): δ = 60.3 (2 CH), 33.4 (2 CH_2), 26.6 (CH_2), 23.4 (2 CH_2) ppm.
The spectroscopic values are in accordance with literature values.[2]

1,2-Dibromoctane (5c):

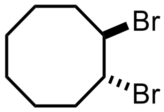

According to GPA, cyclooctane (55 mg, 0.50 mmol, 1.0 equiv.) and tetrabutylammonium bromide (645 mg, 2.00 mmol, 4.0 equiv.) were converted to furnish product **5c** (57 mg, 0.21 mmol, 41%, 82% CE) as colourless oil after column chromatography (SiO_2, *n*-pentane).

^{1}H NMR (500 MHz, $CDCl_3$): δ = 4.63-4.55 (m, 2 H), 2.47-2.37 (m, 2 H), 2.14-2.05 (m, 2 H), 1.91-1.79 (m, 2 H), 1.74-1.56 (m, 4 H), 1.52-1.43 (m, 2 H) ppm. **^{13}C NMR** (125 MHz, $CDCl_3$): δ = 61.7 (2 CH), 33.4 (2 CH_2), 26.1 (2 CH_2), 25.6 (2 CH_2). ppm.
The spectroscopic values are in accordance with literature values.[2]

(1,2-Dibromoethyl)benzene (5d):

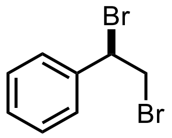

According to GPA, styrene (52 mg, 0.50 mmol, 1.0 equiv.) and tetrabutylammonium bromide (645 mg, 2.00 mmol, 4.0 equiv.) were converted to furnish product **5d** (74 mg, 0.28 mmol, 56%, 112% CE) as colourless oil after column chromatography (SiO_2, *n*-pentane).

^{1}H NMR (500 MHz, $CDCl_3$): δ = 7.46-7.33 (m, 5 H), 5.15 (dd, *J* = 10.6 Hz, 5.4 Hz, 1 H), 4.11-4.01 (m, 2 H) ppm. **^{13}C NMR** (125 MHz, $CDCl_3$): δ = 138.8 (C), 129.3 (CH_{ar}), 129.0 (2 CH_{ar}), 127.8 (CH_{ar}), 51.0 (CH), 35.2 (CH_2) ppm.
The spectroscopic values are in accordance with literature values.[2]

1,2-Dibromo-2,3-dihydro-1*H*-indene (5e):

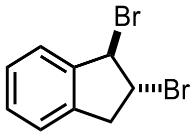

According to GPA, indene (58 mg, 0.50 mmol, 1.0 equiv.) and tetrabutylammonium bromide (645 mg, 2.00 mmol, 4.0 equiv.) were converted to furnish product **5e** (105 mg, 0.38 mmol, 76%, 152% CE) as colourless oil after column chromatography (SiO_2, *n*-pentane).

^{1}H NMR (500 MHz, $CDCl_3$): δ = 7.50-7.46 (m, 1 H), 7.37-7.30 (m, 3 H), 5.64 (s, 1 H), 4.89 (dt, *J* = 5.3, 1.2 Hz, 1 H), 3.82 (dd, *J* = 17.5, 5.3 Hz, 1 H), 3.28 (d, *J* = 17.5 Hz, 1 H) ppm. **^{13}C NMR** (125 MHz, $CDCl_3$): δ = 140.8 (C), 140.7 (C), 129.8 ($CH_{ar.}$), 128.1 ($CH_{ar.}$), 125.9 ($CH_{ar.}$), 125.5 ($CH_{ar.}$), 57.9 (CH), 54.6 (CH), 41.6 (CH_2) ppm.
The spectroscopic values are in accordance with literature values.[2]

1,2-Dibromodecane (5f):

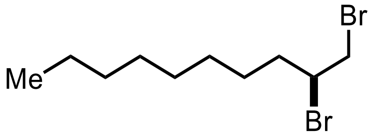

According to GPA, 1-decene (70 mg, 0.50 mmol, 1.0 equiv.) and tetrabutylammonium bromide (645 mg, 2.00 mmol, 4.0 equiv.) were converted to furnish product **5f** (125 mg, 0.415 mmol, 83%, 166% CE) as colourless oil after column chromatography (SiO_2, *n*-pentane).

^{1}H NMR (500 MHz, $CDCl_3$): δ = 4.21-4.13 (m, 1 H), 3.84 (dd, *J* = 10.2, 4.5 Hz, 1 H), 3.63 (t, *J* = 10.0 Hz, 1 H), 2.18-2.09 (m, 1 H), 1.84-1.74 (m, 1 H), 1.63-1.53 (m, 1 H), 1.43-1.25 (m,

11 H), 0.92-0.87 (m, 3 H) ppm. **^{13}C NMR** (125 MHz, $CDCl_3$): δ = 53.3 (CH), 36.5 (CH_2), 36.2 (CH_2), 32.0 (CH_2), 29.5 (CH_2), 29.3 (CH_2), 29.0 (CH_2), 26.9 (CH_2), 22.8 (CH_2), 14.2 (CH_3) ppm. The spectroscopic values are in accordance with literature values.[3]

5,6-Dibromodecane (5g):

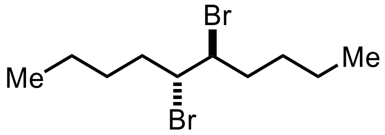

According to GPA, (*E*)-5-decene (70 mg, 0.50 mmol, 1.0 equiv.) and tetrabutylammonium bromide (645 mg, 2.00 mmol, 4.0 equiv.) were converted to furnish product **5g** (147 mg, 0.490 mmol, 98%, 196% CE) as colourless oil after column chromatography (SiO_2, *n*-pentane).

^{1}H NMR (500 MHz, $CDCl_3$): δ = 4.20-4.12 (m, 2 H), 2.17-2.07 (m, 2 H), 2.00-1.89 (m, 2 H), 1.66-1.55 (m, 2 H), 1.48-1.28 (m, 6 H), 0.93 (t, J = 7.2 Hz, 6 H) ppm. **^{13}C NMR** (125 MHz, $CDCl_3$): δ = 60.2 (2 CH), 36.8 (2 CH_2), 29.3 (2 CH_2), 22.2 (2 CH_2), 14.0 (2 CH_3) ppm.
The spectroscopic values are in accordance with literature values.[3]

1,2-Dibromo-2,3,3-trimethylbutane (5h):

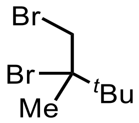

According to GPA, 2,3,3-trimethylbut-1-ene (49 mg, 0.50 mmol, 1.0 equiv.) and tetrabutylammonium bromide (645 mg, 2.00 mmol, 4.0 equiv.) were converted to furnish product **5h** (85 mg, 0.33 mmol, 66%, 132% CE) as colourless oil after column chromatography (SiO_2, *n*-pentane).

^{1}H NMR (300 MHz, $CDCl_3$): δ = 4.03 (d, J = 11.2 Hz, 1 H), 3.88 (d, J = 11.2 Hz, 1 H), 1.88 (s, 3 H), 1.21 (s, 9 H) ppm. **^{13}C NMR** (125 MHz, $CDCl_3$): δ = 79.7 (C), 43.5 (CH_2), 40.6 (C), 27.7 (CH_3), 27.0 (CH_3) ppm. **IR** (ATR): λ^{-1} = 2972 (m), 1469 (m), 1456 (m), 1399 (m), 1377 (s), 1367 (s), 1257 (m), 1237 (m), 1223 (w), 1202 (w), 1193 (w), 1150 (w), 1094 (m), 1077 (w), 1051 (s), 1003 (m), 929 (w), 909 (w), 871 (m), 826 (m), 731 (m), 697 (w), 664 (m), 589 (s), 547 (m) cm^{-1}. **HRMS** (EI, 70 eV): calcd. 255.9457 (for $C_7H_{14}Br_2^+$), found 255.9468 [M^+].

2,3-Dibromo-2,3-dimethylbutane (5i):

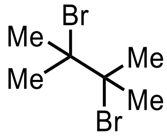

According to GPA, 1,2,3,4-tetramethylethylene (42 mg, 0.50 mmol, 1.0 equiv.) and tetrabutylammonium bromide (645 mg, 2.00 mmol, 4.0 equiv.) were converted to furnish product **5i** (122 mg, 0.500 mmol, 100%, 200% CE) as colourless oil after column chromatography (SiO_2, *n*-pentane).

^{1}H NMR (300 MHz, $CDCl_3$): δ = 2.03 (s, 12 H) ppm. **^{13}C NMR** (125 MHz, $CDCl_3$): δ = 74.1 (2 C), 32.0 (4 CH_3) ppm.
The spectroscopic values are in accordance with literature values.[2]

(3*R*)-6,7-3,7-Dimethyloct-1-ene (5j):

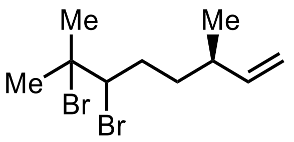

According to GPA, *β*-citronellene (69 mg, 0.50 mmol, 1.0 equiv.) and tetrabutylammonium bromide (645 mg, 2.00 mmol, 4.0 equiv.) were converted to furnish product **5j** (116 mg, 0.389 mmol, 78%, 156% CE) as colourless oil after column chromatography (SiO_2, *n*-pentane).

^{1}H NMR (500 MHz, $CDCl_3$): δ = 5.78-5.63 (m, 1 H), 5.05-4.92 (m, 2 H), 4.18 (dq, J = 5.7, 1.5 Hz, 1 H), 2.51-2.33 (m, 1 H), 2.23-2.14 (m, 1 H), 1.98 (s, 3 H), 1.80 (d, J = 2.4 Hz, 3 H), 1.79-1.66 (m, 2 H), 1.48-1.37 (m, 1 H), 1.04 (dq, J = 6.7, 4.1 Hz, 3 H) ppm. **^{13}C NMR** (125 MHz, $CDCl_3$): δ = 144.3 (CH), 143.9 (CH), 113.6 (CH_2), 113.1 (CH_2), 69.0 (C), 67.2 (CH), 37.4 (CH_3), 35.5 (CH_3), 35.3 (CH_2), 33.9 (CH_2), 33.8 (CH_2), 28.4 (CH_3), 28.3 (CH_3), 20.9 (CH_3), 20.1 (CH_3) ppm.
The spectroscopic values are in accordance with literature values.[4]

1,2-Diiodocycohexane (5k):

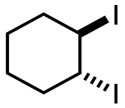

According to GPA, cyclohexene (41 mg, 0.50 mmol, 1.0 equiv.) and tetrabutylammonium iodide (645 mg, 2.00 mmol, 4.0 equiv.) were converted to furnish product **5k** (141 mg, 0.42 mmol, 84%, 168% CE) as colourless oil after column chromatography (SiO_2, *n*-pentane).

^{1}H NMR (300 MHz, $CDCl_3$): δ = 4.53-4.35 (m, 2 H), 2.52-2.39 (m, 2 H), 1.94-1.73 (m, 4 H), 1.58-1.42 (m, 2 H), 1.37-1.18 (m, 2 H), 0.94-0.80 (m, 2 H) ppm. **^{13}C NMR** (125 MHz, $CDCl_3$): δ = 55.3 (2 CH), 32.2 (2 CH_2), 22.5 (2 CH_2) ppm.
The spectroscopic values are in accordance with literature values.[5]

7,8-Dibromooct-1-ene (5l):

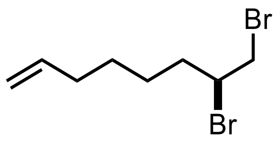

According to GPA, 1,7-octadiene (55 mg, 0.50 mmol, 1.0 equiv.) and tetrabutylammonium bromide (645 mg, 2.00 mmol, 4.0 equiv.) were converted to furnish product **5l** (52 mg, 0.19 mmol, 38%) as colourless oil after column chromatography (SiO_2, *n*-pentane). In the course of this reaction, 1,2,7,8-tetrabromooctene (**5m**, 55%) was generated too hence the current efficiency assembles of both yields: conversion of the educt: 92%, 147% CE.

^{1}H NMR (500 MHz, $CDCl_3$): δ = 5.90-5.75 (m, 1 H), 5.07-4.91 (m, 2 H), 4.21-4.12 (m, 1 H), 3.85 (dd, J = 10.2, 4.4 Hz, 1 H), 3.63 (t, J = 9.9 Hz, 1 H), 2.22-2.04 (m, 3 H), 1.89-1.73 (m, 1 H), 1.65-1.54 (m, 1 H), 1.51-1.38 (m, 3 H) ppm. **^{13}C NMR** (125 MHz, $CDCl_3$): δ = 138.6 (CH), 114.9 (CH_2), 53.1 (CH), 36.4 (CH_2), 36.1 (CH_2), 33.6 (CH_2), 28.2 (CH_2), 26.4 (CH_2) ppm.
IR (ATR, neat): λ^{-1} = 2934 (m), 2859 (w), 1640 (w), 1460 (w), 1431 (m), 1374 (w), 1267 (w), 1224 (m), 1142 (m), 991 (m), 911 (m), 733 (m), 644 (m), 566 (s) cm^{-1}. **HRMS** (EI, 70 eV): calcd. 267.9457 (for $C_8H_{14}Br_2^+$), found 267.9465 [M^+].

1,2,7,8-Tetrabromooctene (5m):

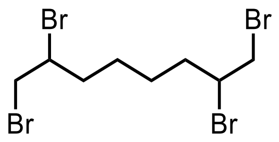

According to GPA, 1,7-octadiene (55 mg, 0.50 mmol, 1.0 equiv.) and tetrabutylammonium bromide (645 mg, 2.00 mmol, 4.0 equiv.) were converted to furnish product **5m** (116 mg, 0.270 mmol, 55%) as colourless oil after column chromatography (SiO_2, *n*-pentane). In the course of this reaction, 7,8-dibromooct-1-ene (**5l**, 38%) was generated too, hence the current efficiency assembles of both yields: conversion of the educt: 92%, 147% CE.

^{1}H NMR (500 MHz, $CDCl_3$): δ = 4.22-4.23 (m, 2 H), 3.86 (dd, J = 10.3, 4.4 Hz, 2 H), 3.63 (t, J = 10.1 Hz, 2 H), 2.25-2.12 (m, 2 H), 1.89-1.77 (m, 2 H), 1.72-1.41 (m, 4 H) ppm. **^{13}C NMR** (125 MHz, $CDCl_3$): δ = 53.70 (CH), 52.68 (CH), 36.26 (CH_2), 36.25 (CH_2), 35.93 (CH_2), 35.85 (CH_2), 26.2 (CH_2), 26.1 (CH) ppm. **IR** (ATR): λ^{-1} = 2939 (m), 2860 (w), 1460 (w), 1432 (m),

1264 (m), 1216 (m), 1142 (m), 1050 (w), 989 (w), 899 (w), 754 (s), 737 (s), 704 (m), 667 (m), 644 (m), 566 (s) cm^{-1}. **HRMS** (EI, 70 EV): calcd. 425.7824 (for $C_8H_{14}Br_4^+$), found 425.7816 [M^+].

1-Bromo-2,4-dimethoxybenzene (9):

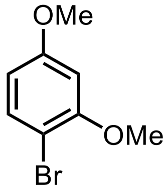

According to GPA, 1,3-dimethoxybenzene (**8**, 69 mg, 0.50 mmol, 1.0 equiv.) and tetrabutylammonium bromide (645 mg, 2.00 mmol, 4.0 equiv.) were electrolyzed for 1.2 *F* to furnish product **9** (101 mg, 0.465 mmol, 94%, 168% CE) as colourless oil after column chromatography (SiO_2, *n*-pentane).

^{1}H NMR (300 MHz, $CDCl_3$): δ = 7.40 (d, J = 8.7 Hz, 1 H), 6.48 (d, J = 2.8 Hz, 1 H), 6.40 (dd, J = 8.7, 2.8 Hz, 1 H), 3.87 (s, 3 H), 3.80 (s, 3 H) ppm. **^{13}C NMR** (125 MHz, $CDCl_3$): δ = 160.4 (C), 156.8 (C), 133.3 (CH), 106.1 (CH), 102.7 (CH), 100.2 (CH), 56.3 (CH_3), 55.7 (CH_3) ppm. The spectroscopic values are in accordance with literature values.[6]

2.5. Robustness Screen

The robustness screen was done according to literature procedures of GLORIUS.[7-8]

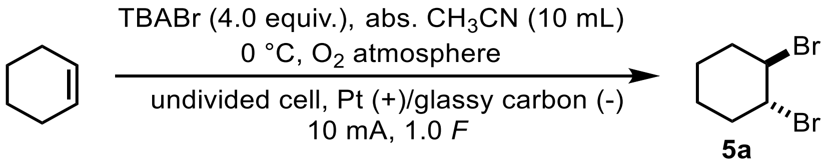

5a

First, the tetrabutylammonium bromide (TBABr) (645 mg, 2.00 mmol, 4.0 equiv.) was weighed into an undivided cell and dissolved in abs. acetonitrile (10 mL). Afterwards, water (0.90 mL, 90 mg, 5.0 mmol, 10.0 equiv.), conc. sulfuric acid (0.11 mL, 196 mg, 2.00 mmol, 4.0 equiv.), cyclohexene (41 mg, 0.50 mmol, 1.0 equiv.) and the corresponding additive (0.50 mmol, 1.0 equiv.) were added and the reaction mixture was cooled to 0 °C with an ice bath. At this temperature, the solution was saturated with oxygen by bubbling the gas through the solution for 10 minutes. Afterwards, the reaction mixture was electrolyzed under constant current (10 mA, 1.0 *F*, Pt cathode, glassy carbon anode) at 0 °C and under oxygen atmosphere. After the electrolysis mesitylene was added as internal standard and the yield of the product and the additive were analyzed by GC-FID analysis of the crude reaction mixture.

Table 5: Robustness Screen using cyclohexene as substrate.[a]

Entry	Additive	Yield (Product 5a) [%][b]	Yield (Additive) [%][b]
1	none	95	---
2	1-dodecine	84	96
3	benzonitrile	75	97
4	octanenitrile	77	96
5	bromobenzene	82 (81)	98 (98)
6	dibutyl ether	72 (74)	83 (81)
7	sulfolane	77	5
8	1-chlorooctane	71 (79)	91 (91)
9	aniline	75	24
10	cyclopropyl benzene	78	99
11	valerophenone	84	79
12	4-bromonitrobenzene	49	67
13	dodecylamine	77 (75)	7 (4)
14	2,6-lutidine	79	0
15	*N*-octanale	59	54
16	1,2-epoxy-*n*-octane	80	66

17	cyclohexanone	15	23
18	1,3-dimethoxybenzene	71	64
19	*N*,*N*-dimethylaniline	74	69
20	acetanilidine	89	53
21	1-octanole	50	72
22	methyl benzoate	87	86

Colour code: green: yield > 66%, yellow: yields between 34-66%, red: yields <34%. [a] All reactions were Performed on a 0.50 mmol scale. The yields in brackets are the ones of retries. [b] The yields were determined by GC analysis using mesitylene as internal standard.

3. Mechanistic Investigations

3.1. Control Experiments

To gain information about the role of sulfuric acid in this reaction, some control experiments were performed. Thereby, cyclohexene should be converted to 1,2-dibromocyclohexane (**5a**) under various conditions (Scheme 1).

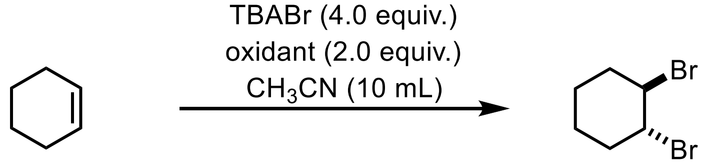

Scheme 1: Conversion of cyclohexene to **5a** under various conditions as control experiments.

Experiment 1: First, tetrabutylammonium bromide (TBABr) (1.29 g, 4.00 mmol, 4.0 equiv.) was weighed into a round bottom flask and dissolved in acetonitrile (10 mL). Afterwards, cyclohexene (82.1 mg, 1.00 mmol, 1.0 equiv.) and a 30% aqueous H_2O_2 solution (0.23 ml, 2.0 mmol, 2.0 equiv.) were added. The reaction mixture was stirred at room temperature for 24 h, but no conversion of the alkene was observed.

Experiment 2: First, tetrabutylammonium bromide (TBABr) (1.29 g, 4.00 mmol, 4.0 equiv.) was weighed into a round bottom flask and dissolved in acetonitrile (10 mL) and sulfuric acid (0.11 mL, 2.0 mmol, 2.0 equiv.) was added. Afterwards, cyclohexene (82.1 mg, 1.00 mmol, 1.0 equiv.) and a 30% aqueous H_2O_2 solution (0.23 ml, 2.0 mmol, 2.0 equiv.) were added. After the reaction mixture has been stirred for 1 h, mesitylene was added as internal standard and the yield of the product was analyzed by GC-FID analysis of the crude reaction mixture. The product **5a** amounted to 97%.

Experiment 3: First, tetrabutylammonium bromide (TBABr) (1.29 g, 4.00 mmol, 4.0 equiv.) was weighed into a round bottom flask and dissolved in acetonitrile (10 mL) and 48% aqueous HBr-solution (0.43 mL, 2.0 mmol, 2.0 equiv.) was added. Afterwards, cyclohexene (82.1 mg, 1.00 mmol, 1.0 equiv.) and a 30% aqueous H_2O_2 solution (0.23 ml, 2.0 mmol, 2.0 equiv.) were added. After the reaction mixture has been stirred for 1 h, mesitylene was added as internal standard and the yield of the product was analyzed by GC-FID analysis of the crude reaction mixture. The product **5a** amounted to >99%.

3.2. Cyclic voltammograms

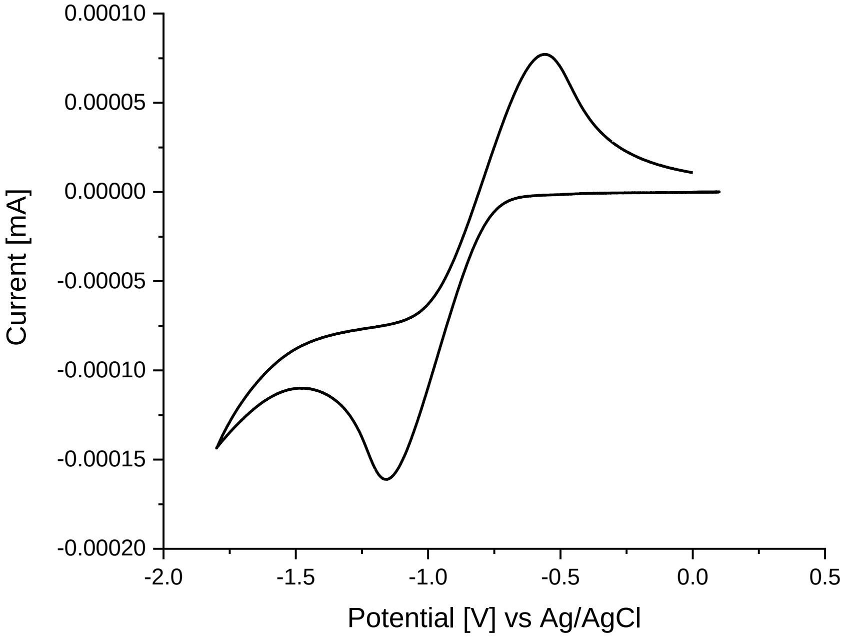

Figure 2: Cyclic voltammogram of an oxygen saturated acetonitrile solution (10 mL) of Bu_4NBr (10 mM) utilising a glassy carbon working electrode, a platinum wire counter electrode and an Ag/AgCl reference electrode (3 M NaCl) at 50 mV·sec^{-1}.

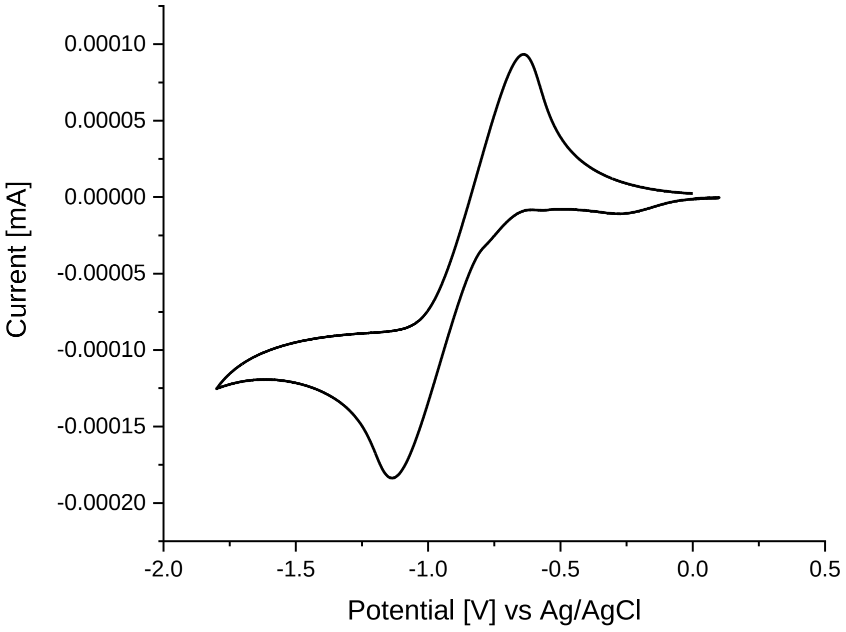

Figure 3: Cyclic voltammogram of an oxygen saturated acetonitrile solution (10 mL) of Bu_4NBr (10 mM) and cyclohexene (2.5 mM) utilising a glassy carbon working electrode, a platinum wire counter electrode and an Ag/AgCl reference electrode (3 M NaCl) at 50 mV·sec^{-1}.

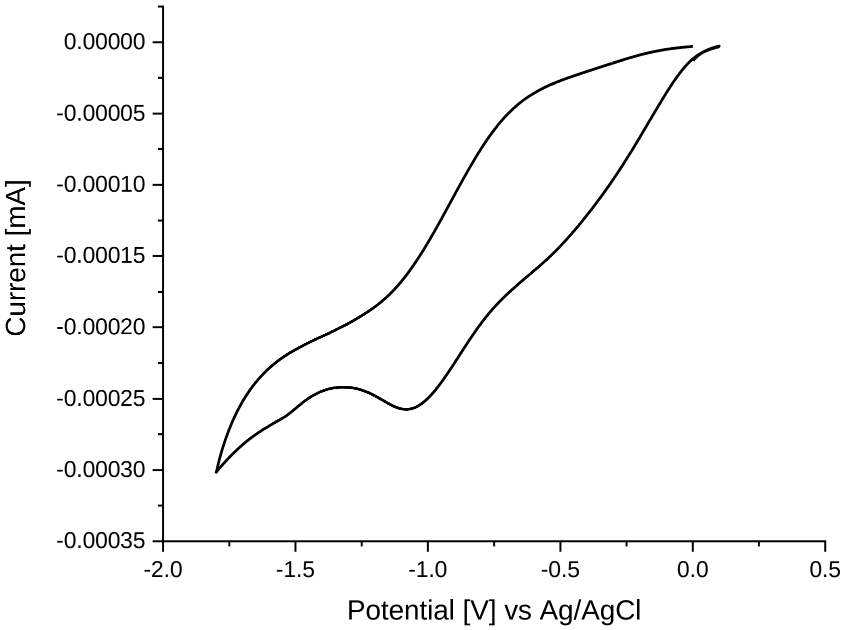

Figure 4: Cyclic voltammogramm of an oxygen saturated acetonitrile solution (10 mL) of Bu_4NBr (10 mM) with H_2SO_4 (c = 0.2 M) utilising a glassy carbon working electrode, a platinum wire counter electrode and an Ag/AgCl reference electrode (3 M NaCl) at 50 mV·sec^{-1}.

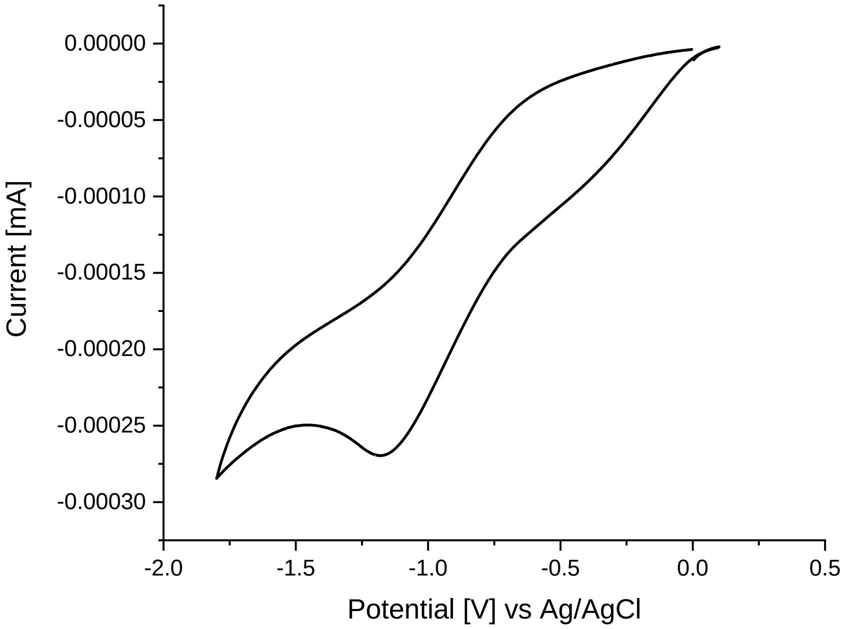

Figure 5: Cyclic voltammogramm of an oxygen saturated acetonitrile solution (10 mL) of Bu_4NBr (10 mM), cyclohexene (2.5 mM) and H_2SO_4 (c = 0.2 M) utilising a glassy carbon working electrode, a platinum wire counter electrode and an Ag/AgCl reference electrode (3 M NaCl) at 50 mV·sec^{-1}.

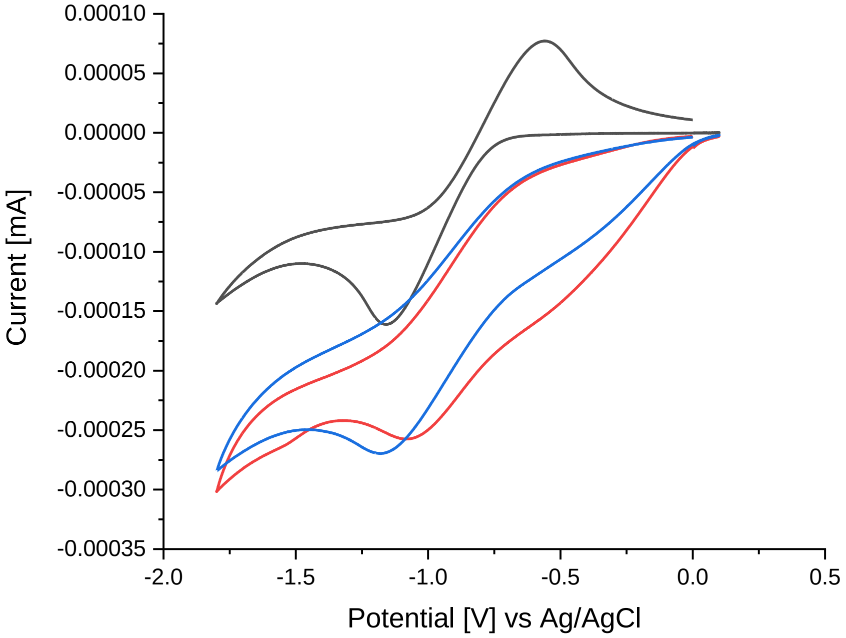

Figure 6: Cyclic voltammogramm of an oxygen saturated acetonitrile solution (10 mL) cyclohexene (2.5 mM) and utilising a glassy carbon working electrode, a platinum wire counter electrode and an Ag/AgCl reference electrode (3 M NaCl) at 50 mV·sec^{-1}. Black curve: nBu_4NBr (10 mM); Red curve: nBu_4NBr (10 mM) and H_2SO_4 (c = 0.2 M); Blue curve: nBu_4NBr (10 mM) and H_2SO_4 (c = 0.2 M) and cyclohexene (2.5 mM).

Cyclic voltammograms for the oxygen reduction on a glassy carbon working electrode showed an quasi-reversible reduction of oxygen (E^{p}_{red} = –1.16 V vs Ag/AgCl) to the superoxid radical-anion ($O_2^{\cdot -}$) under neutral conditions (black curve, Figure 6).[a] This quasi-reversible peak disappears in acidic solution upon protonation and disproportionation towards H_2O_2 and oxygen (red curve) leading to an irreversible cyclic voltammogram.[b] The H_2O_2 reacts under acidic conditions with bromide anions to generate Br_2 and H_2O and in the presence of an alkene (blue curve) a slight peak shift but no dramatic change. Therefore, we propose that the formation of H_2O_2 leads to a fast follow-up formation of Br_2 / Br_3^- and consequent bromination of the alkene.

[a] P. Cofre, D. T. Sawyer, *Anal. Chem.* **1986**, *58*, 1057 – 1062.

[b] C. P. Andrieux, P. Hapiot, J. M. Saveant, *J. Am. Chem. Soc.* **1987**, *109*, 3768 – 3775.

4. NMR spectra of all synthesized compounds

1,2-Dibromocyclohexane (5a):

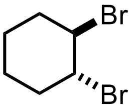

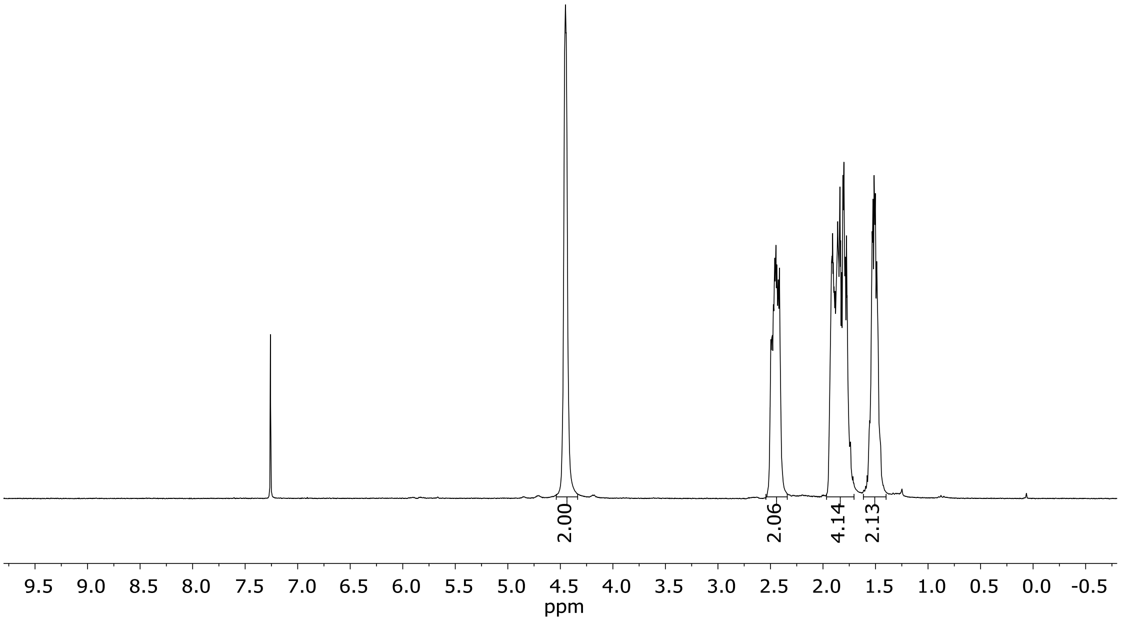

^{1}H NMR (300 MHz, $CDCl_3$) of compound **5a**.

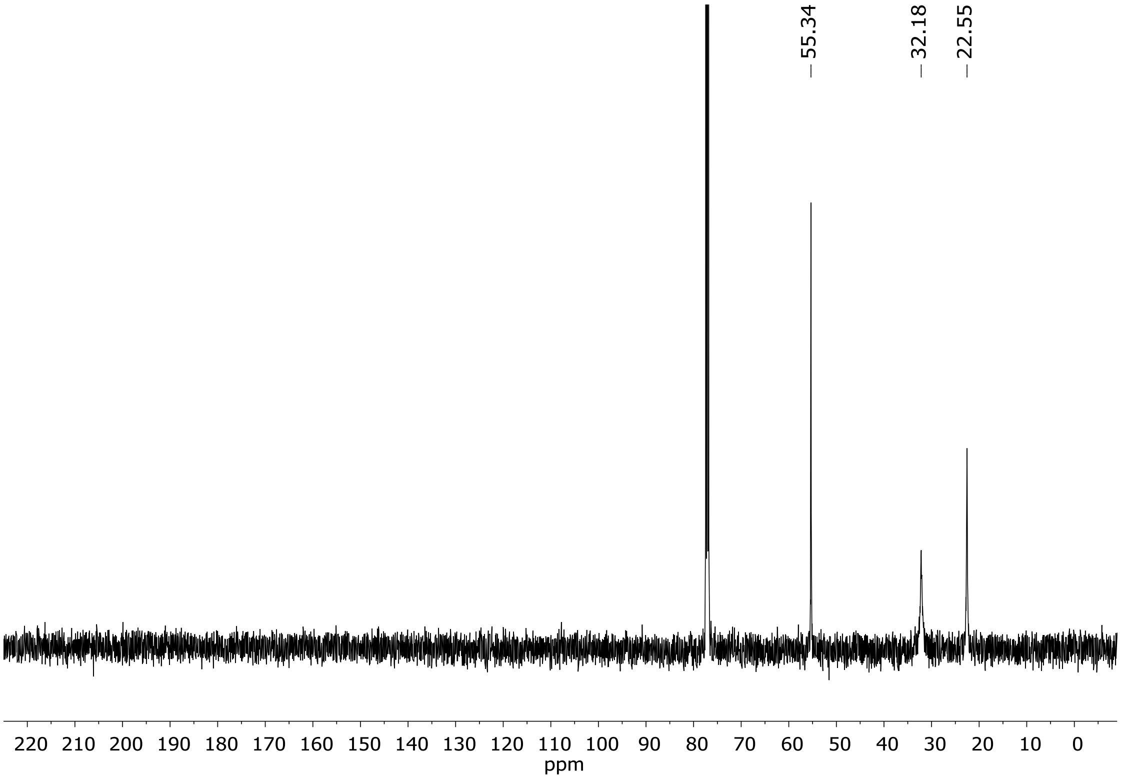

^{13}C NMR (125 MHz, $CDCl_3$) of compound **5a**.

1,2-Dibromoheptane (5b):

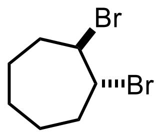

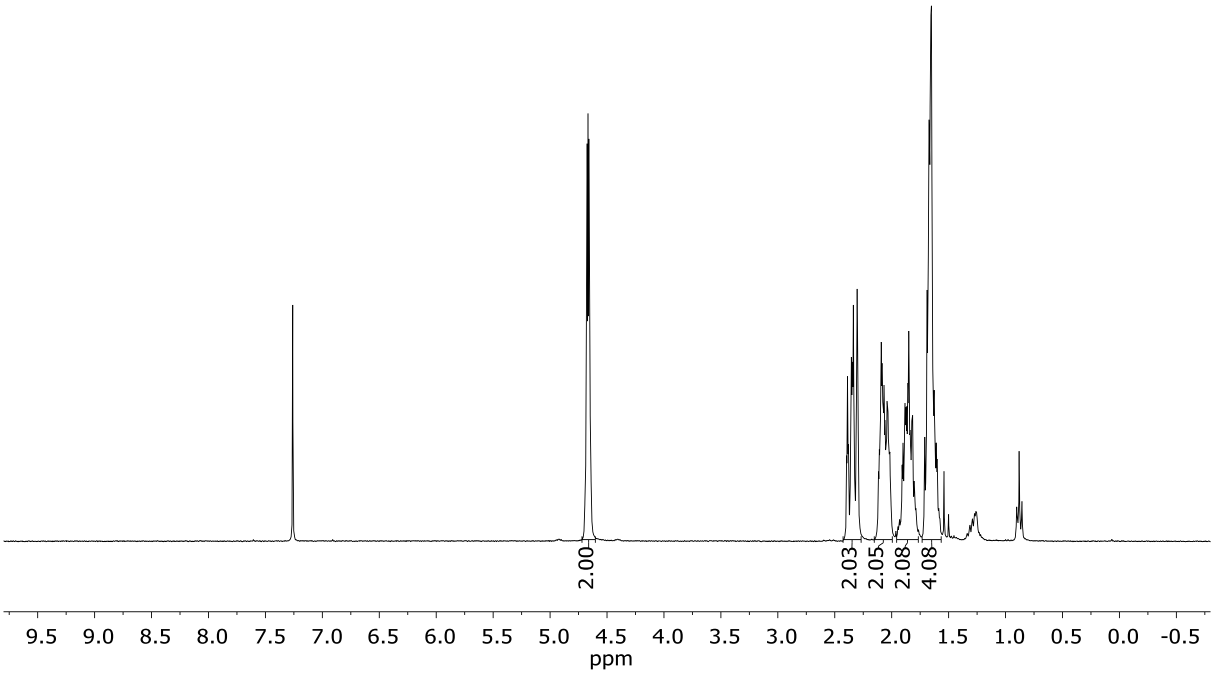

^{1}H NMR (300 MHz, $CDCl_3$) of compound **5b**.

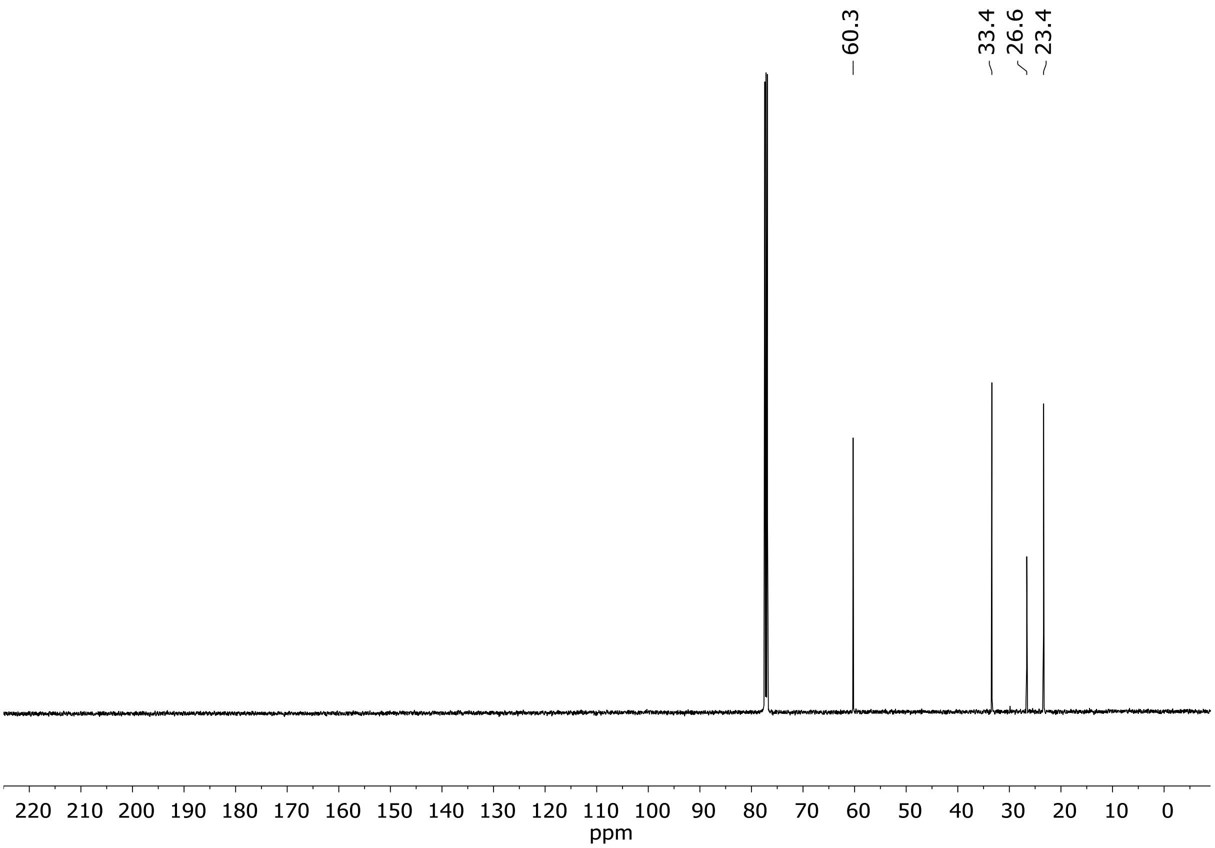

^{13}C NMR (125 MHz, $CDCl_3$) of compound **5b**.

1,2-Dirbomocyclooctane (5c):

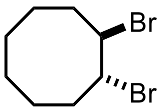

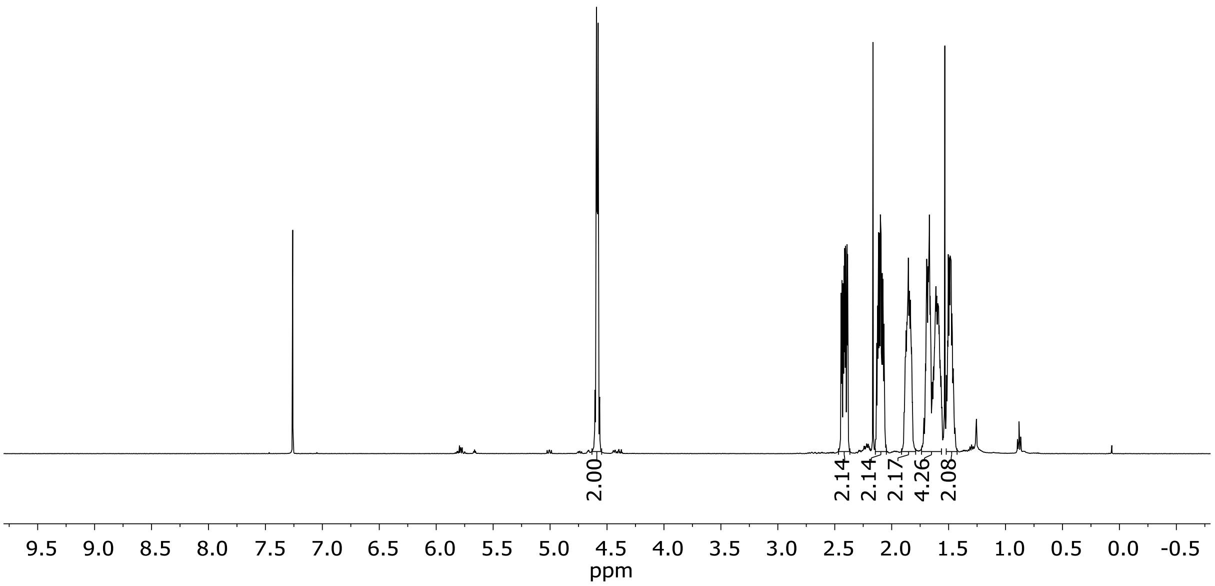

^{1}H NMR (500 MHz, $CDCl_3$) of compound **5c**.

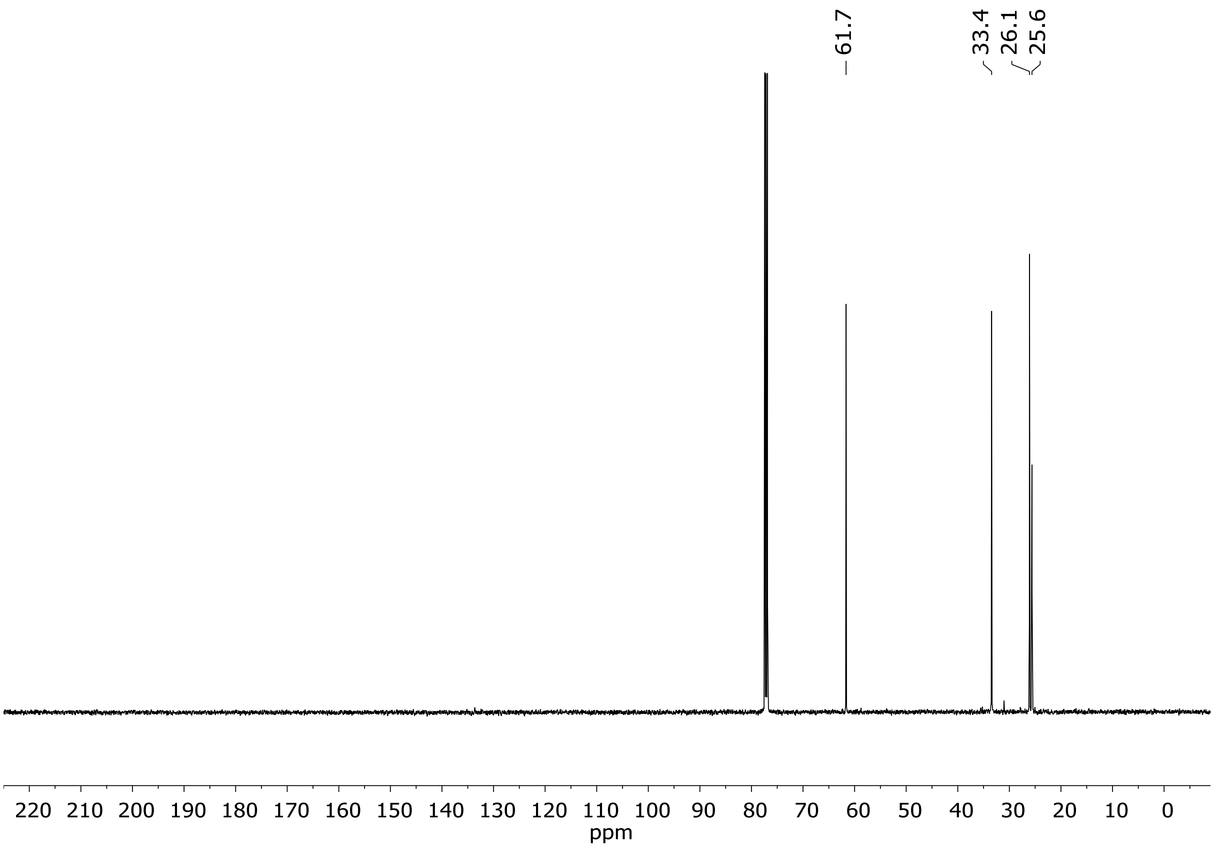

^{13}C NMR (125 MHz, $CDCl_3$) of compound **5c**.

1,2-Dibromoethylbenzene (5d):

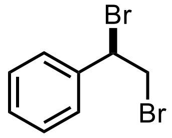

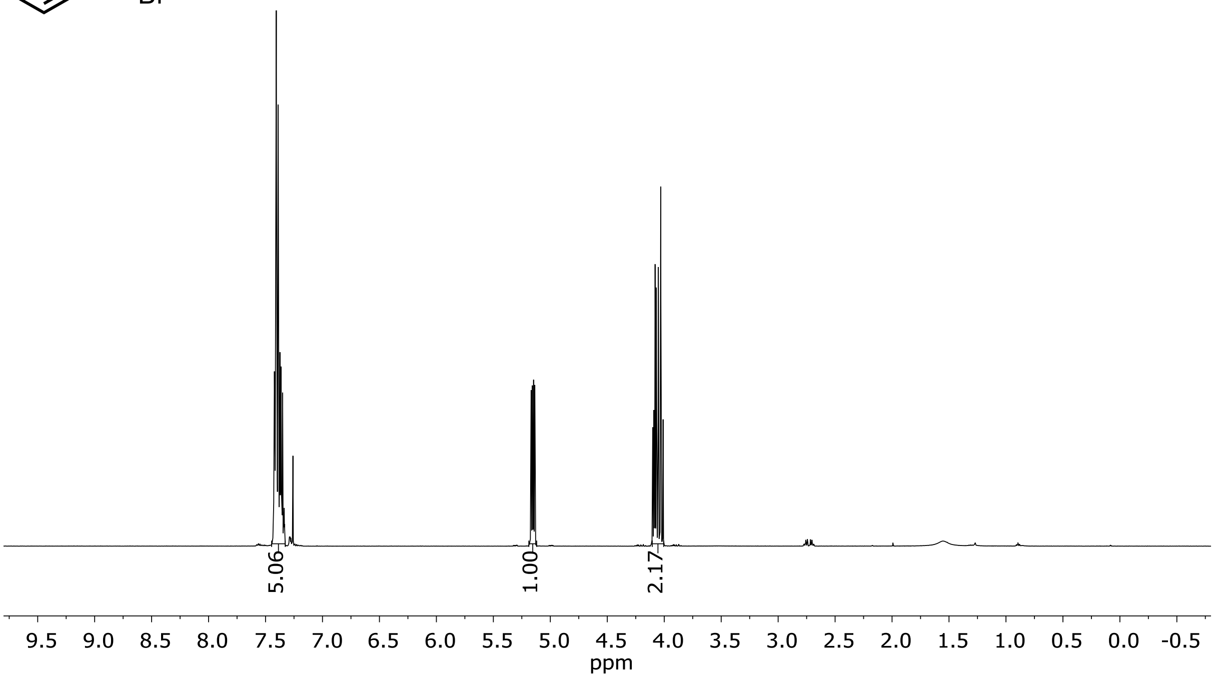

^{1}H NMR (500 MHz, $CDCl_3$) of compound **5d**.

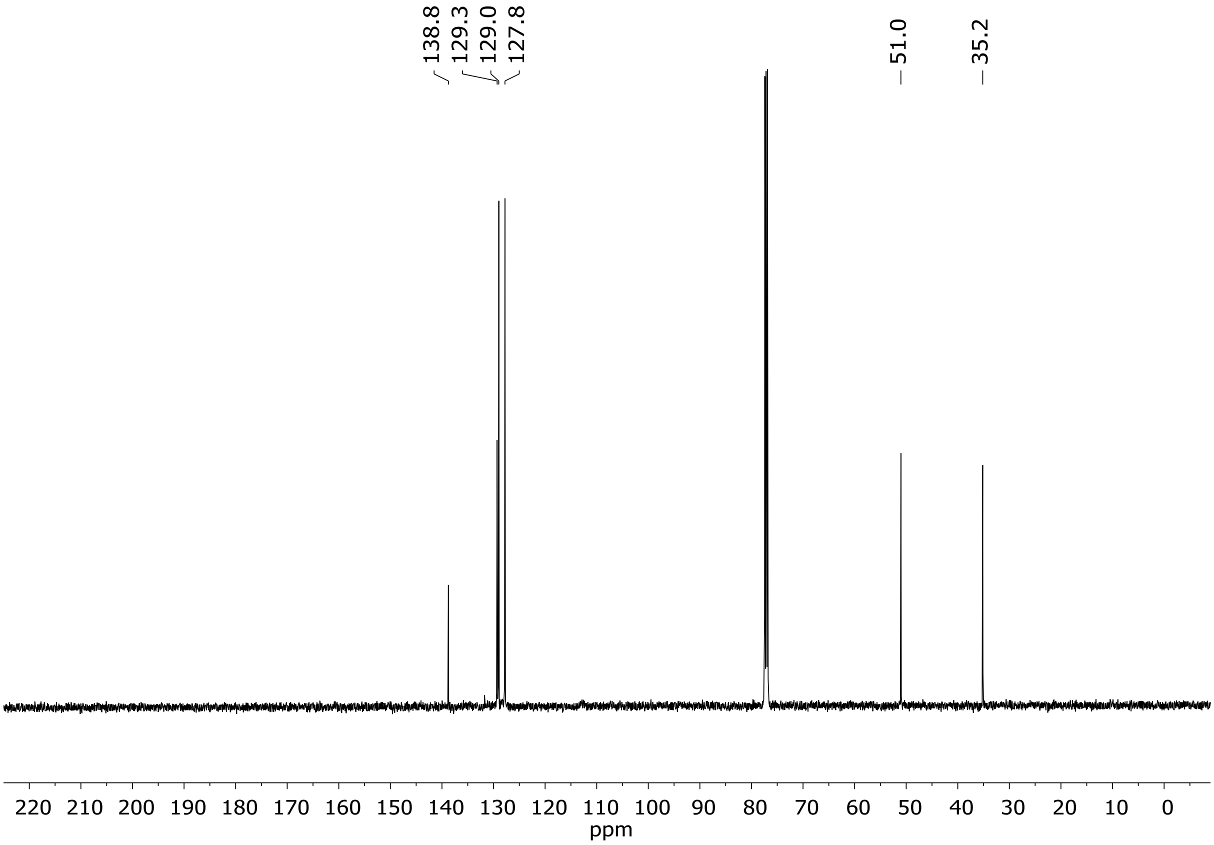

^{13}C NMR (125 MHz, $CDCl_3$) of compound **5d**.

1,2-Dibromo-2,3-dihydro-1*H*-indene (5e):

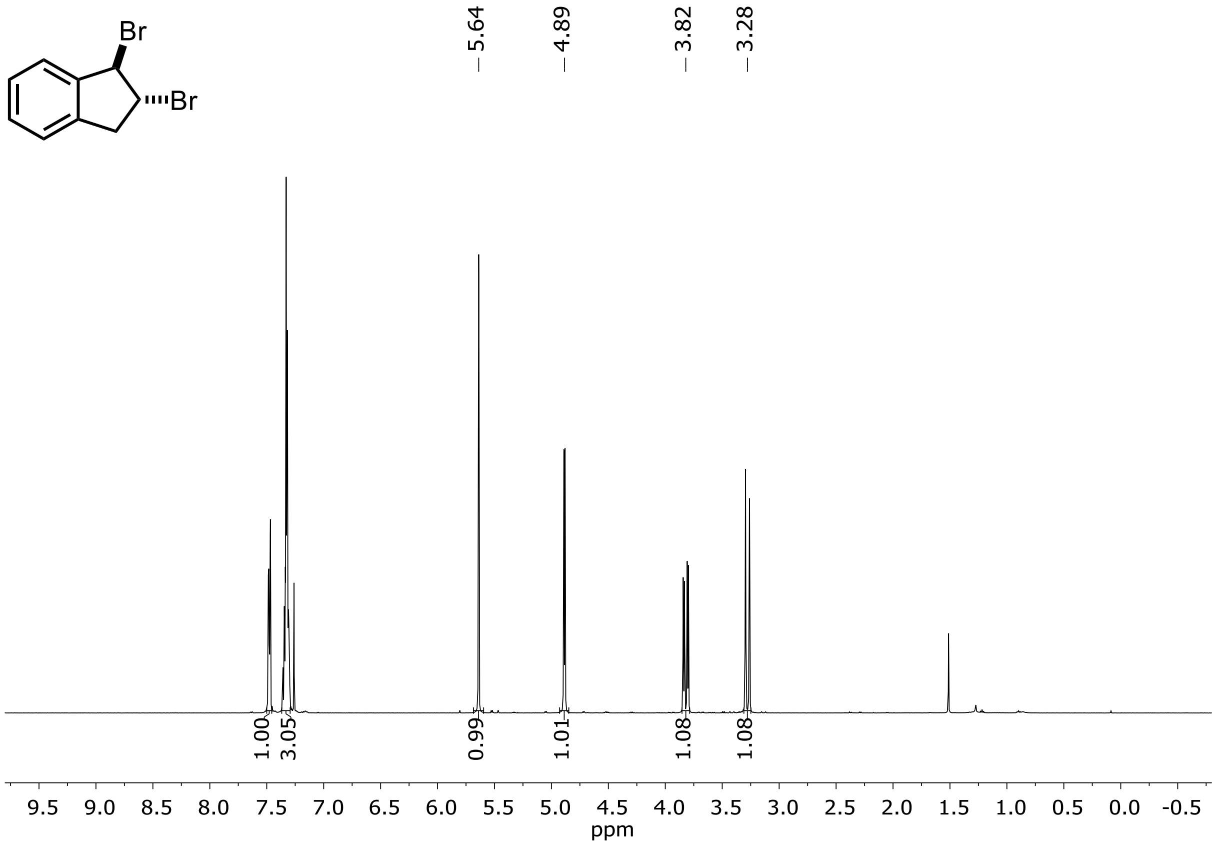

^{1}H NMR (500 MHz, $CDCl_3$) of compound **5e**.

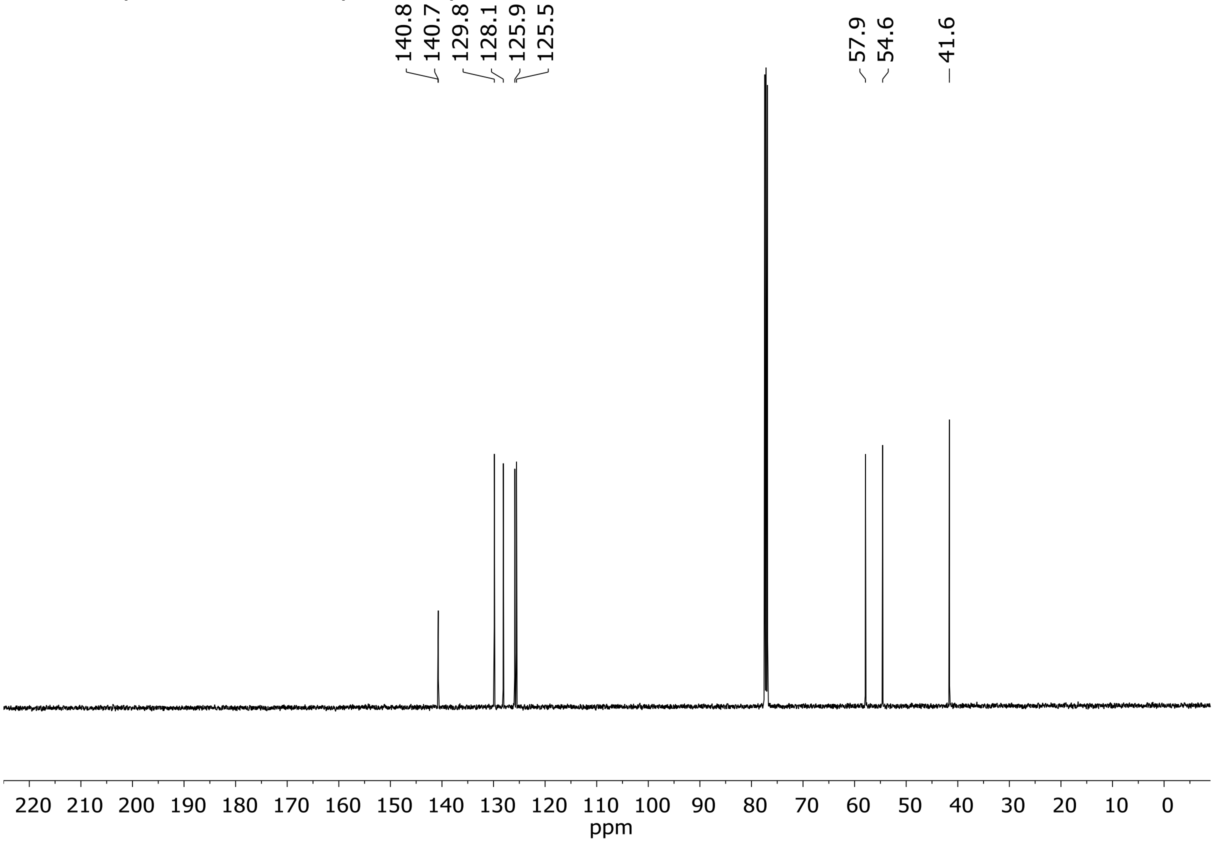

^{13}C NMR (125 MHz, $CDCl_3$) of compound **5e**.

1,2-Dibromodecane (5f):

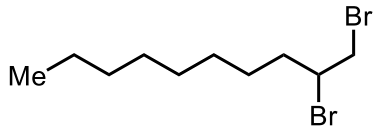

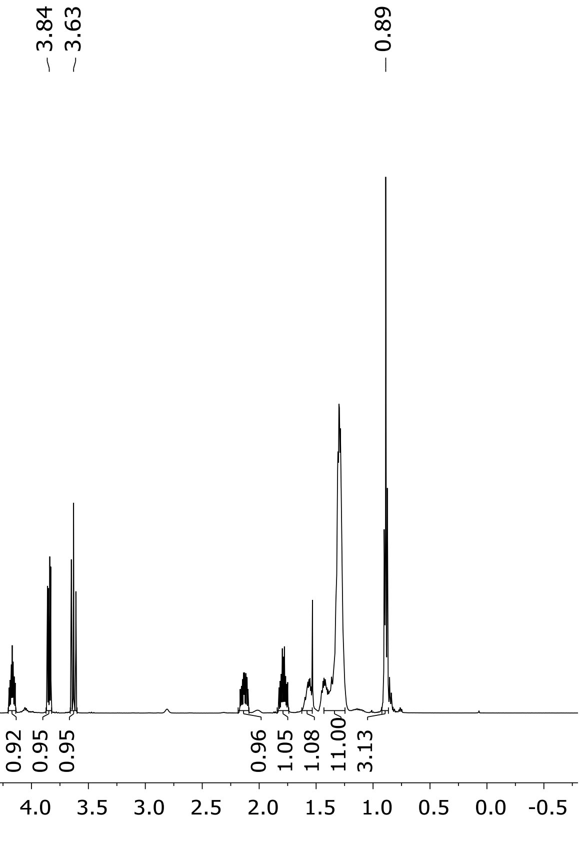

^{1}H NMR (500 MHz, $CDCl_3$) of compound **5f**.

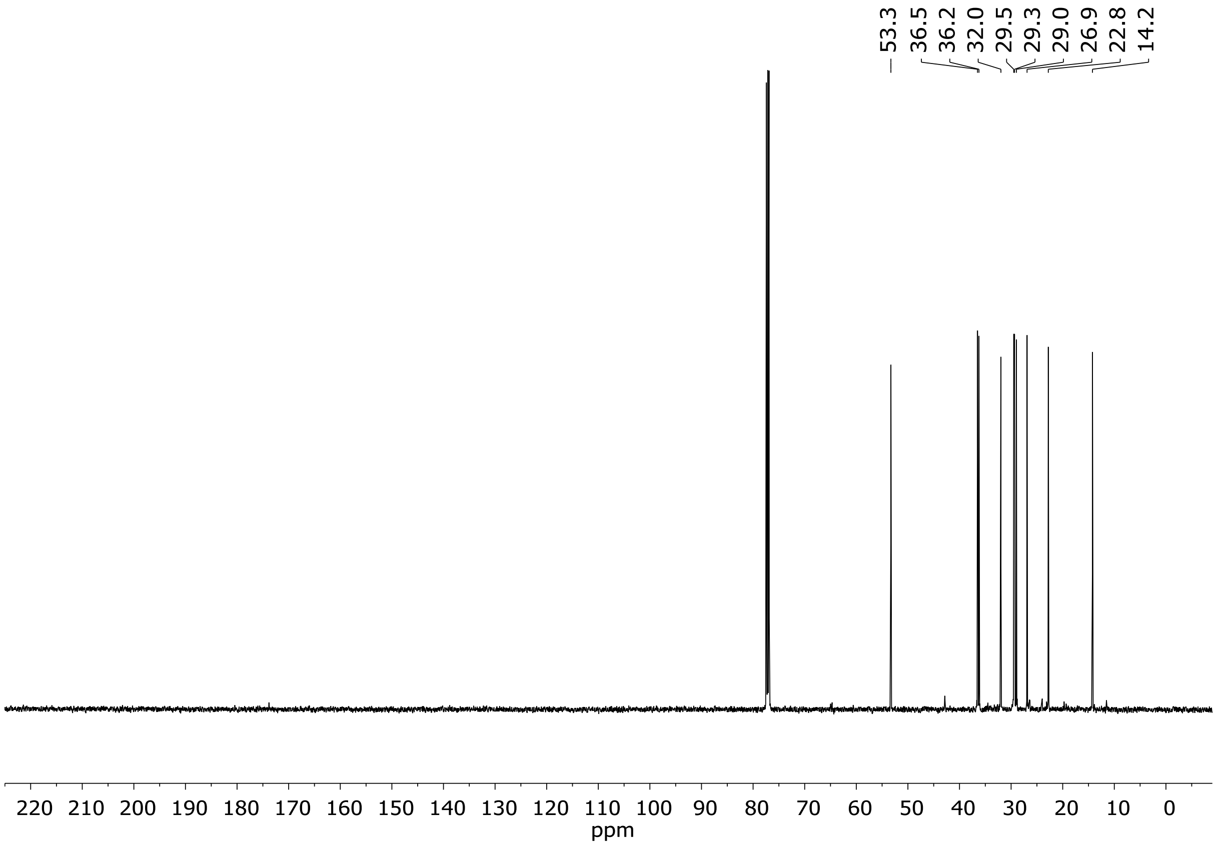

^{13}C NMR (125 MHz, $CDCl_3$) of compound **5f**.

5,6-Dibromodecane (5g):

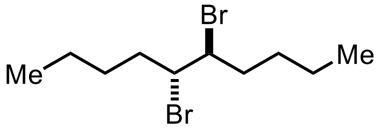

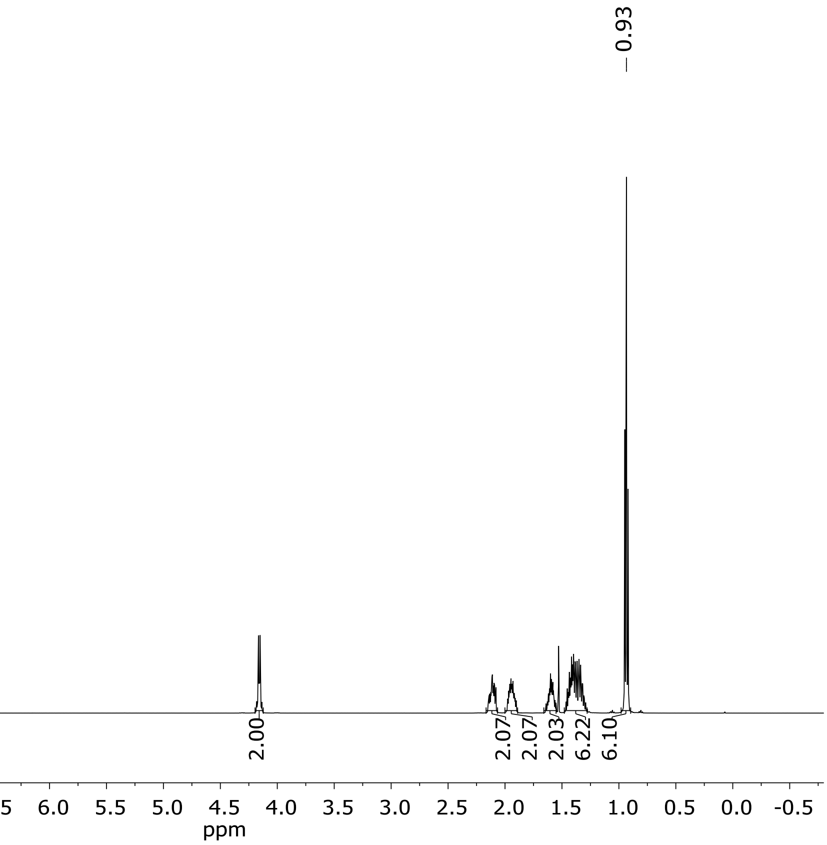

^{1}H NMR (500 MHz, $CDCl_3$) of compound **5g**.

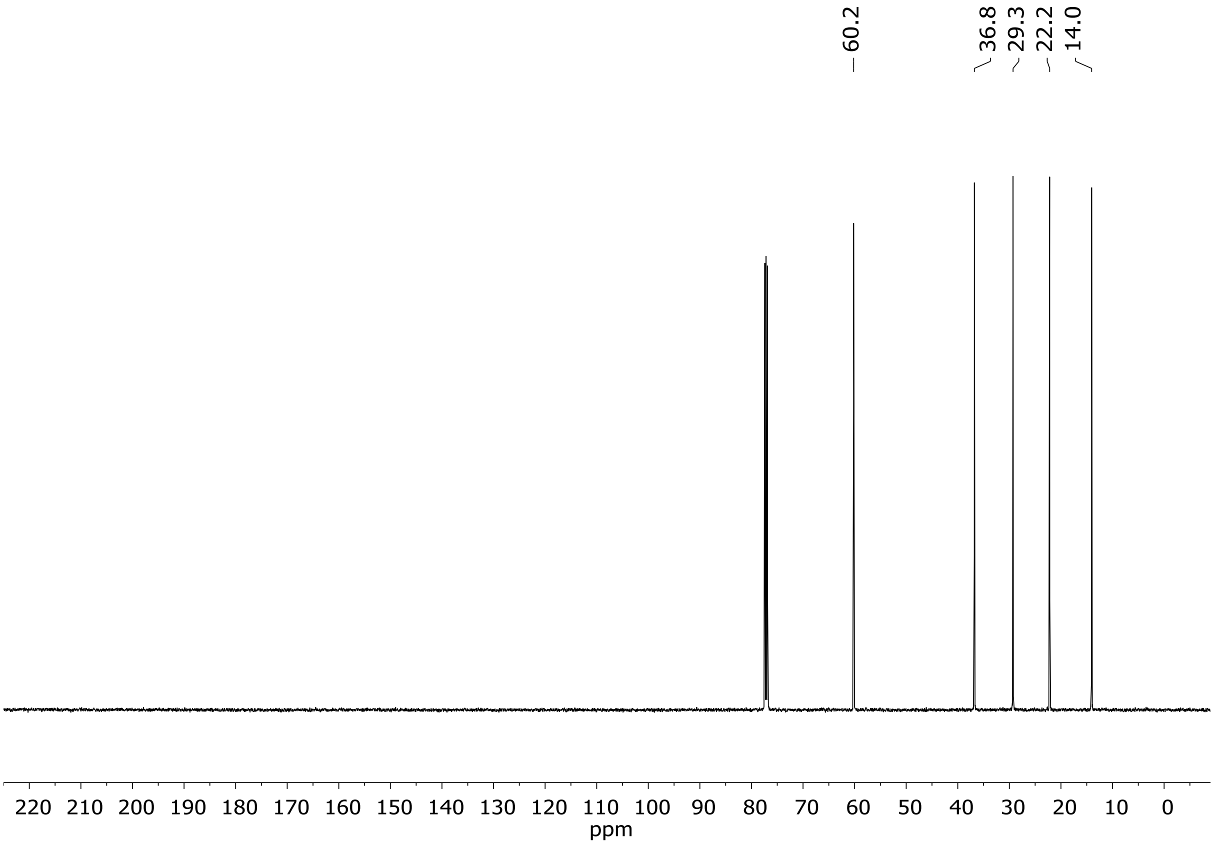

^{13}C NMR (125 MHz, $CDCl_3$) of compound **5g**.

1,2-Dibromo-2,3,3-trimethylbutane (5h):

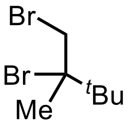

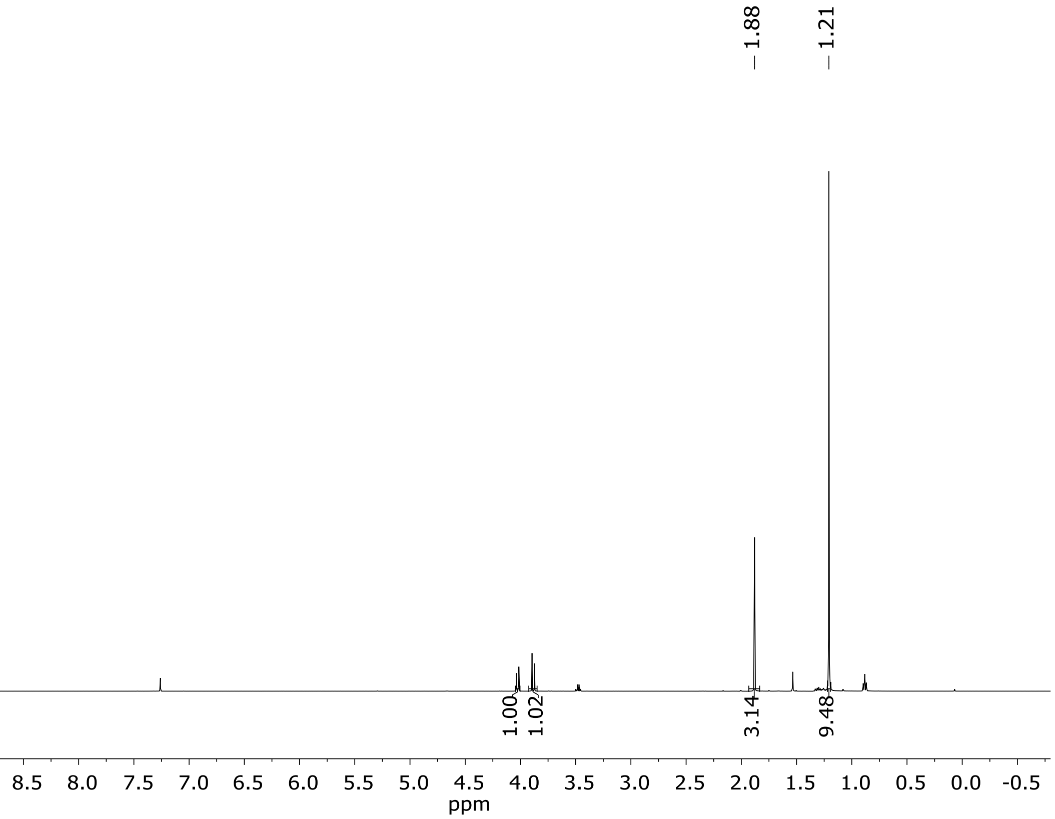

^{1}H NMR (300 MHz, $CDCl_3$) of compound **5h**.

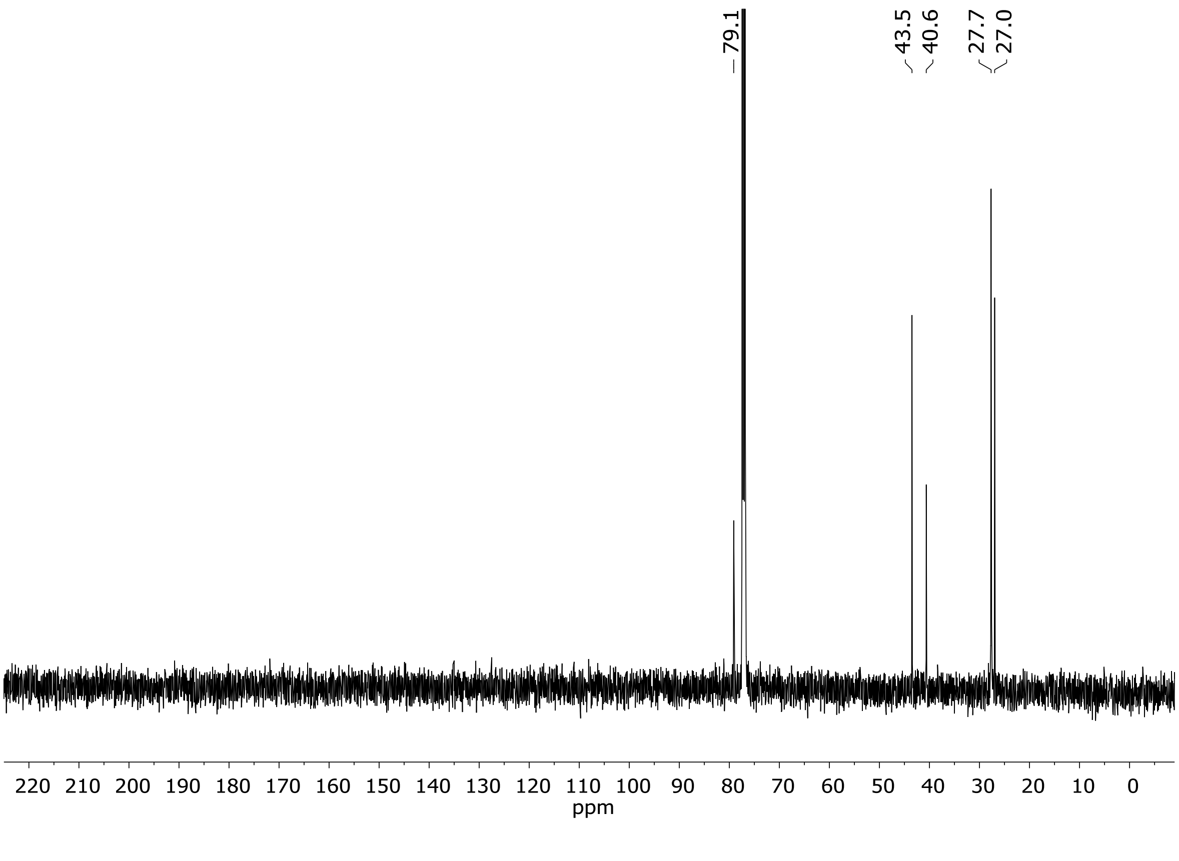

^{13}C NMR (125 MHz, $CDCl_3$) of compound **5h**.

2,3-Dibromo-2,3-dimethylbutane (5i):

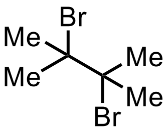

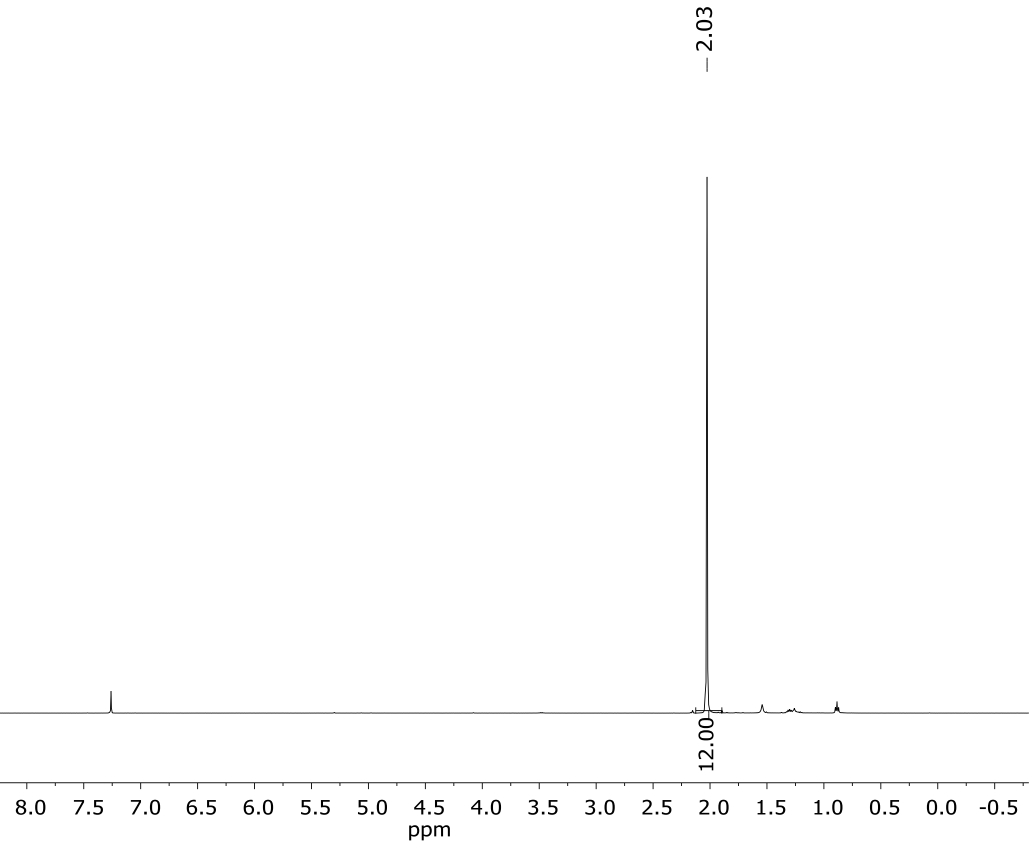

^{1}H NMR (300 MHz, $CDCl_3$) of compound **5i**.

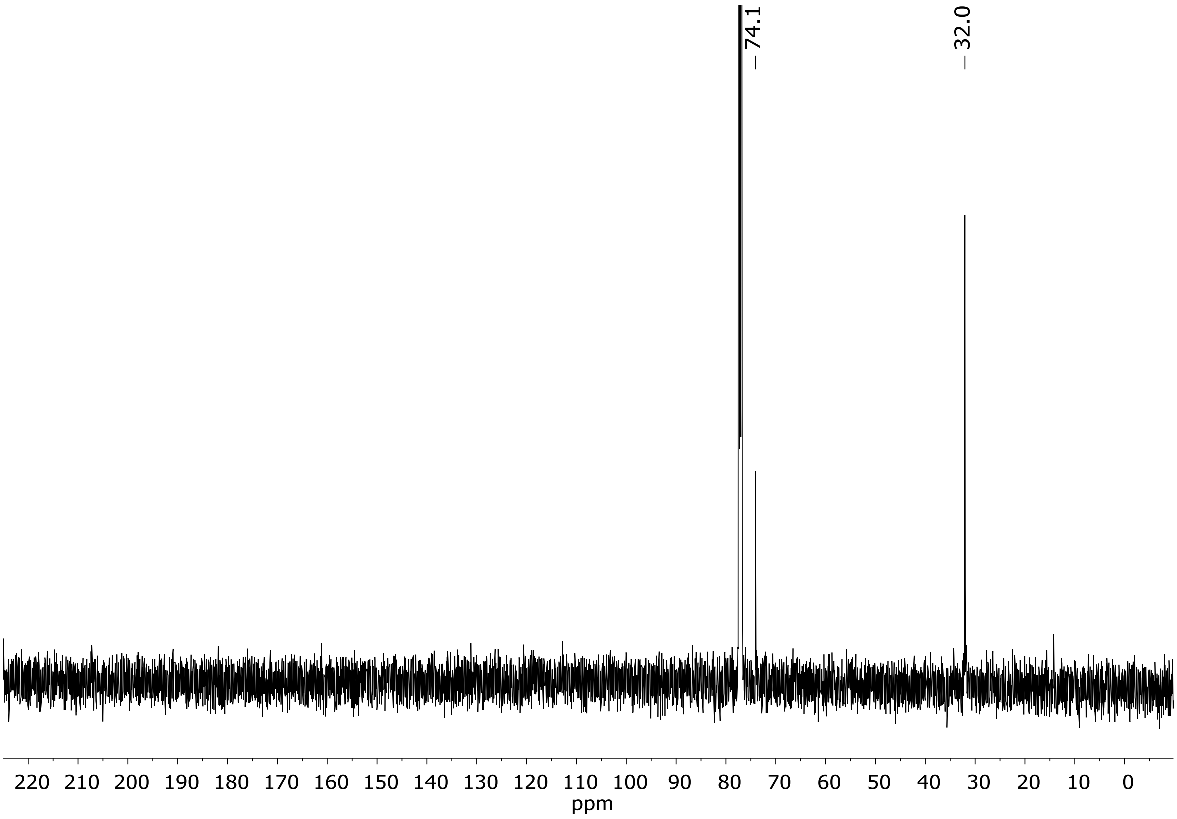

^{13}C NMR (125 MHz, $CDCl_3$) of compound **5i**.

(3*R*)-6,7-3,7-dimethyloct-1-ene (5j):

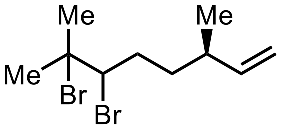

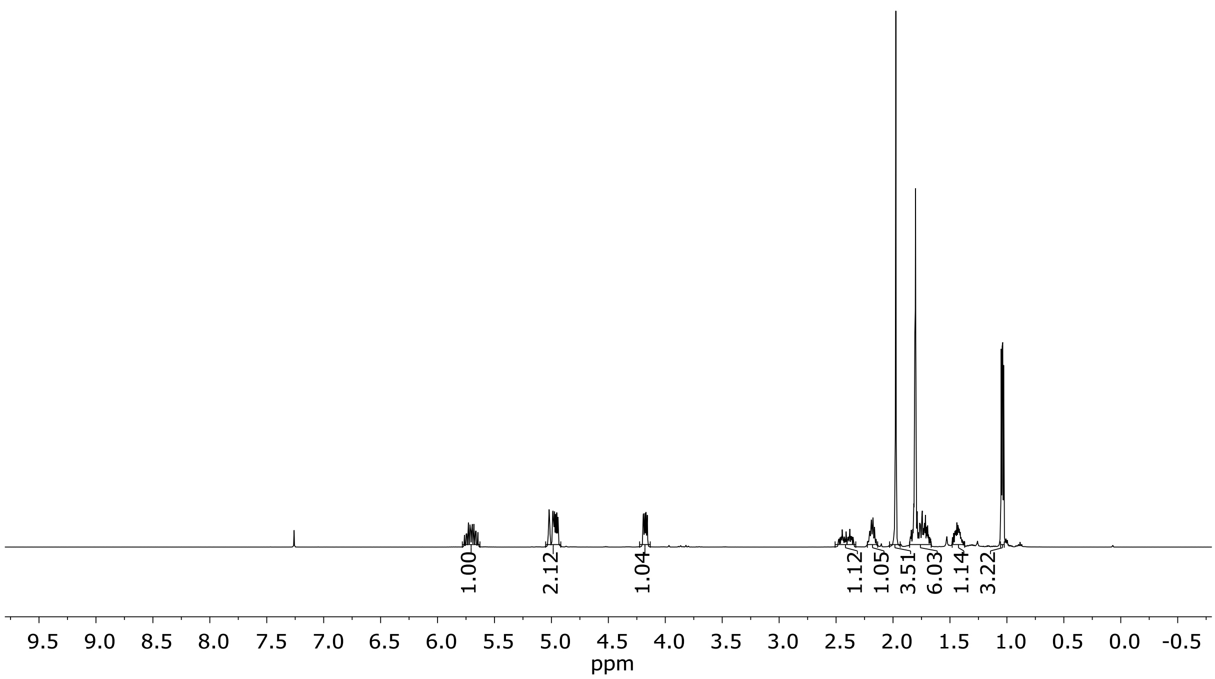

^{1}H NMR (500 MHz, $CDCl_3$) of compound **5j**.

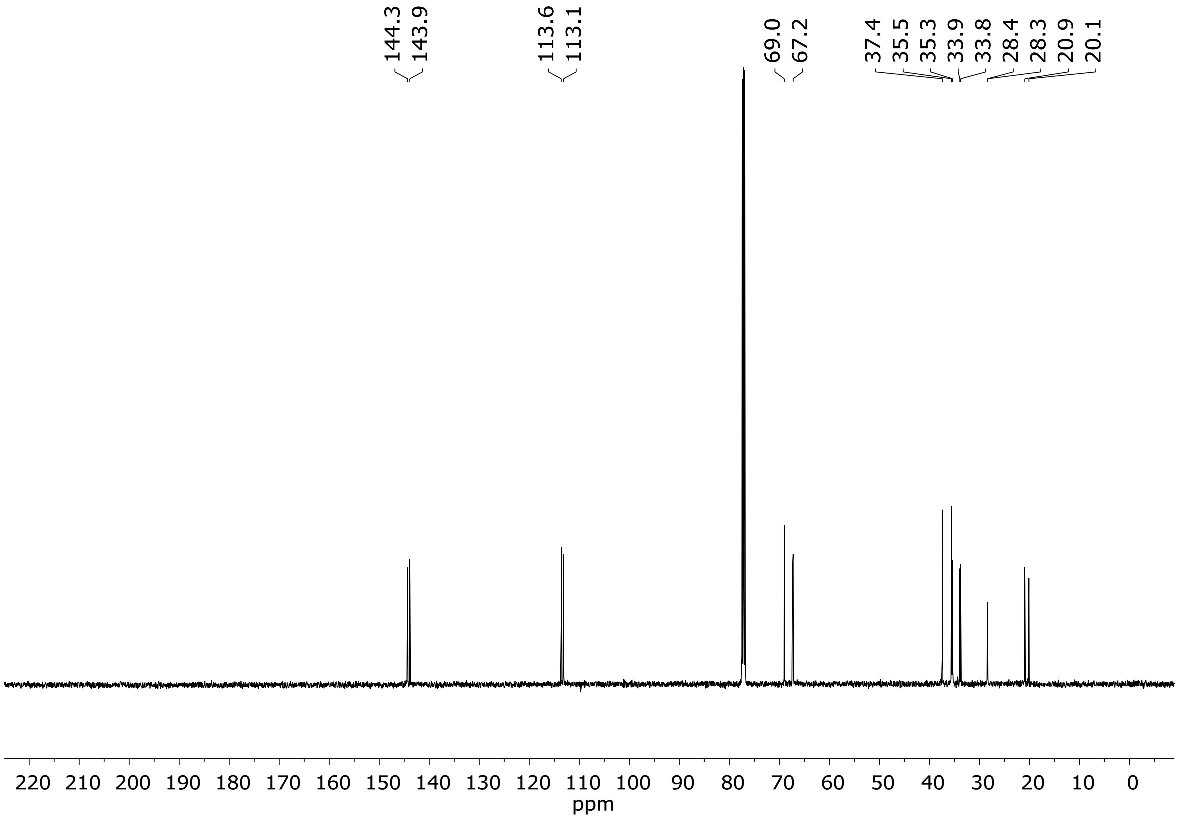

^{13}C NMR (125 MHz, $CDCl_3$) of compound **5j**.

1,2-Diiodocycohexane (5k):

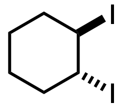

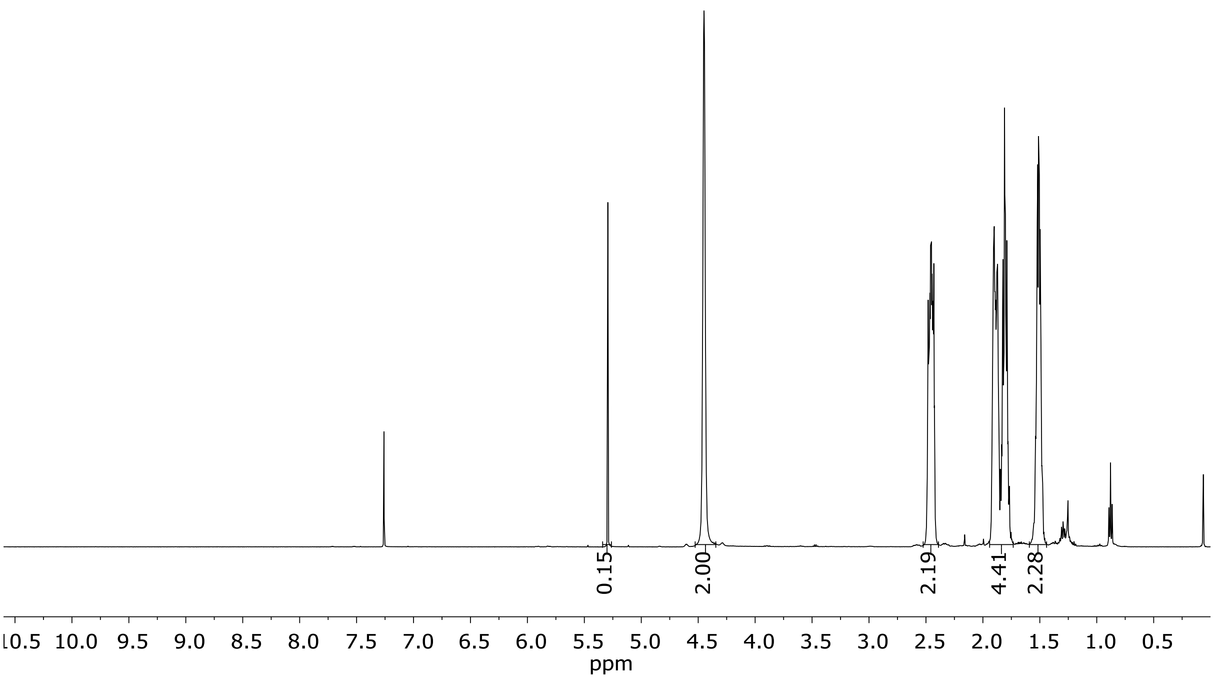

^{1}H NMR (300 MHz, $CDCl_3$) of compound **5k**.

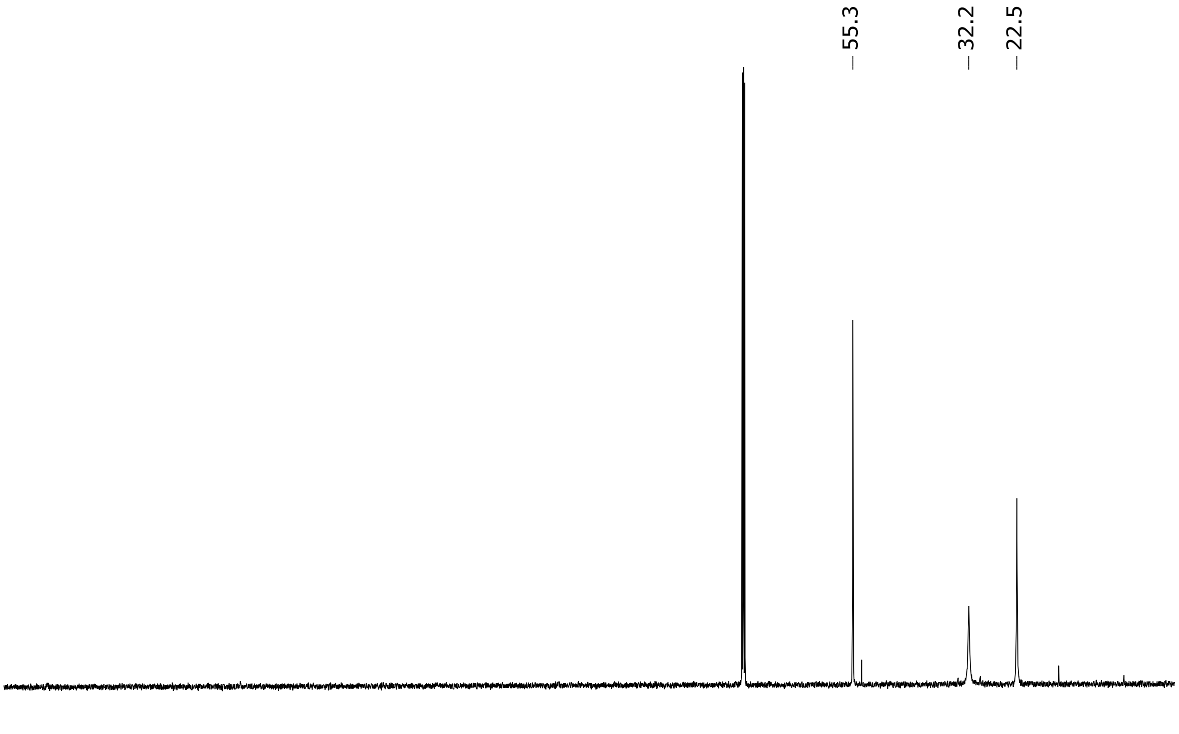

^{13}C NMR (125 MHz, $CDCl_3$) of compound **5k**.

7,8-Dibromooct-1-ene (5l):

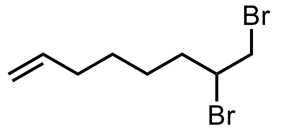

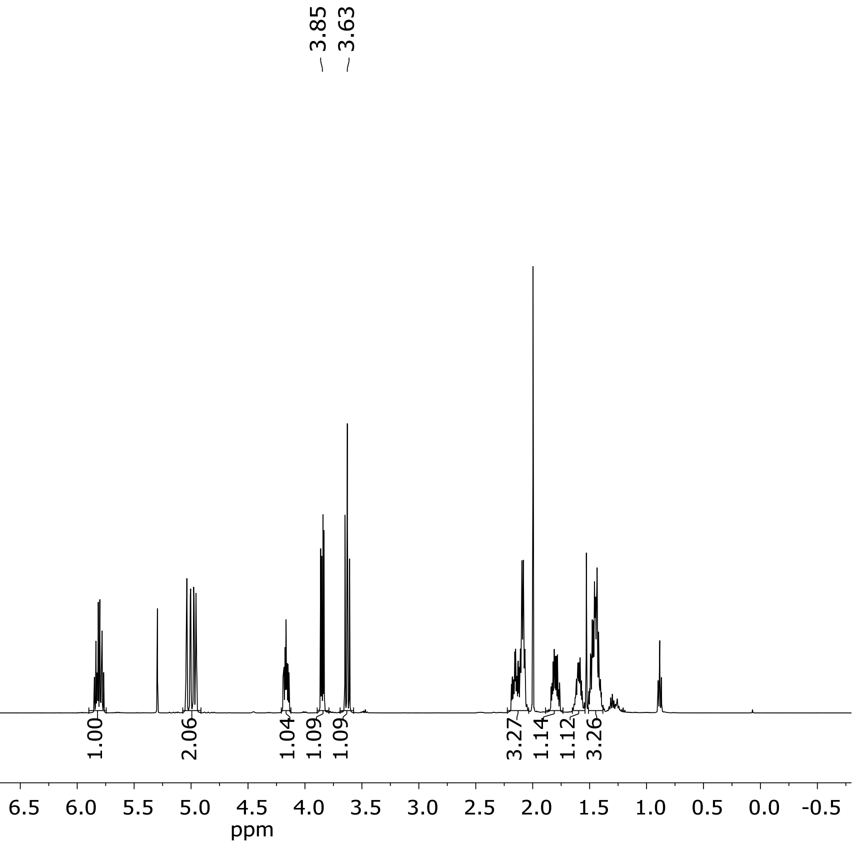

^1H NMR (500 MHz, $CDCl_3$) of compound **5l**.

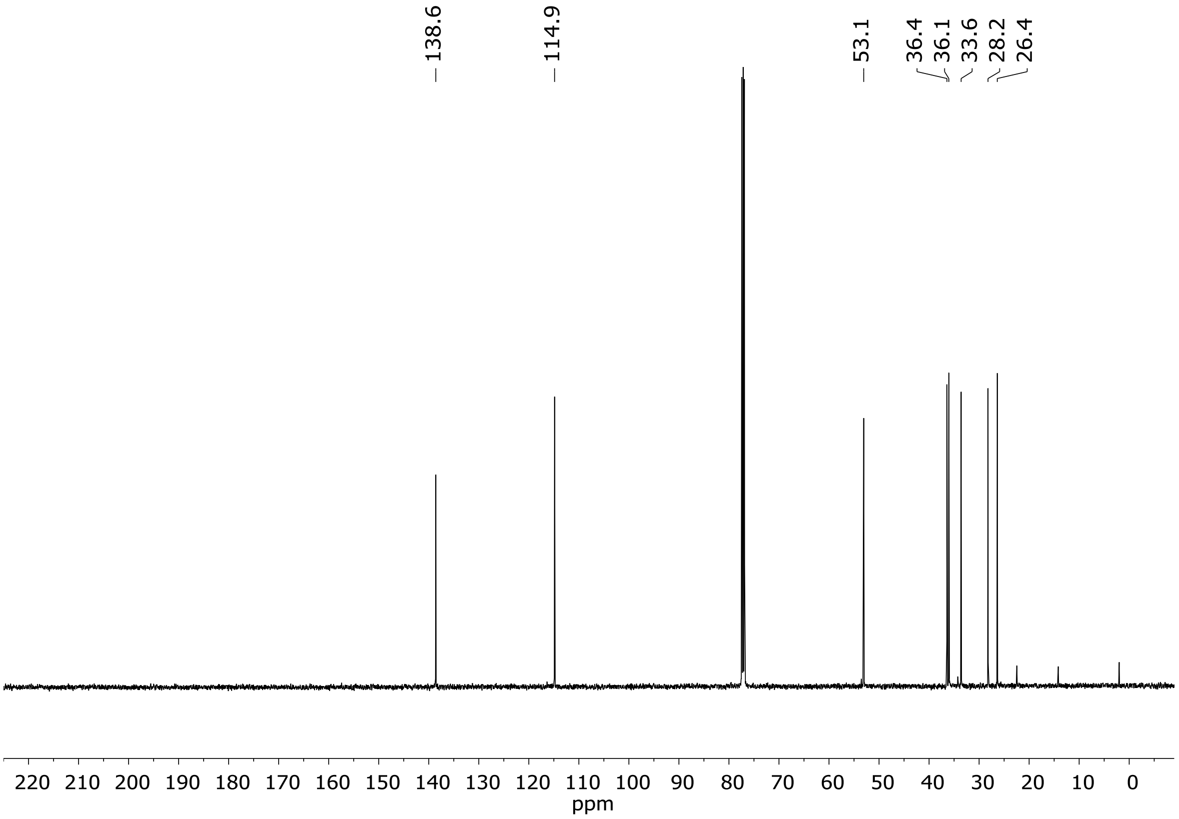

^{13}C NMR (125 MHz, $CDCl_3$) of compound **5l**.

1,2,7,8-Tetrabromooctene (5m):

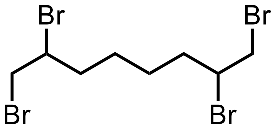

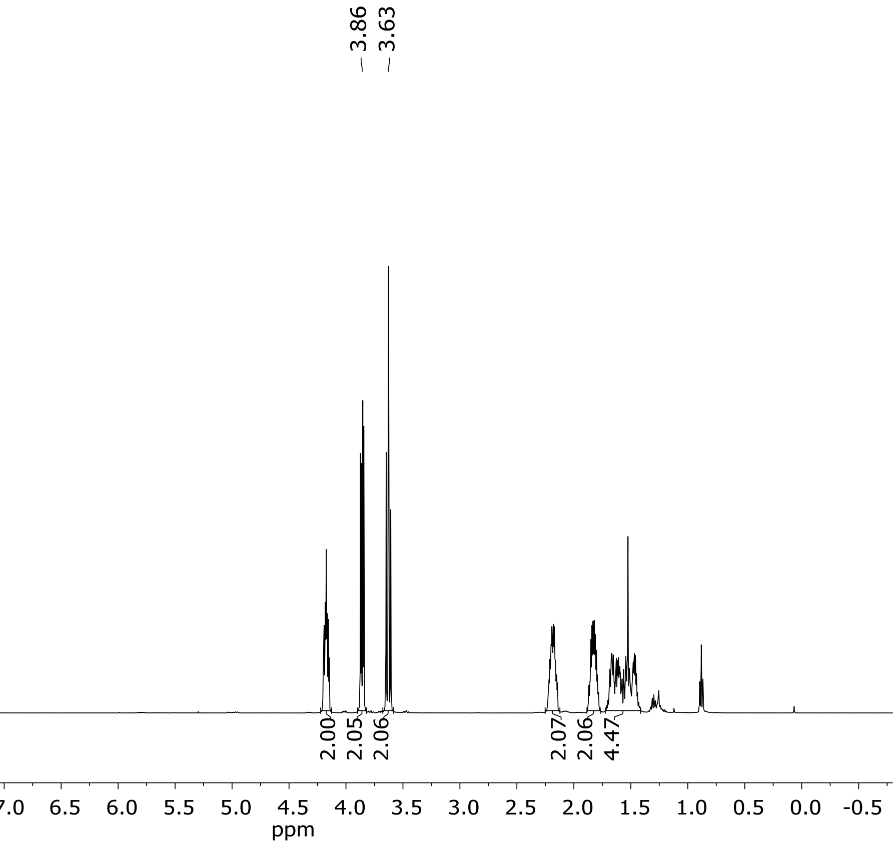

^{1}H NMR (500 MHz, $CDCl_3$) of compound **5m**.

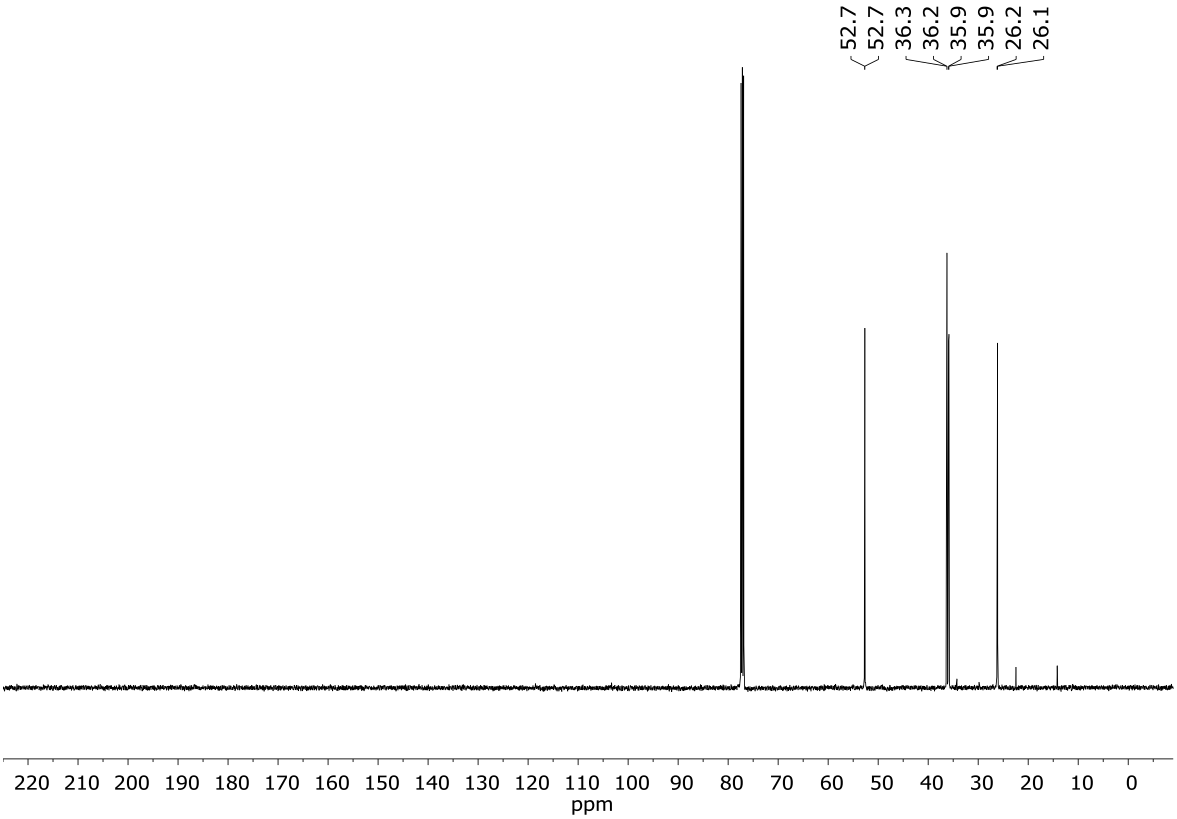

^{13}C NMR (125 MHz, $CDCl_3$) of compound **5m**.

1-Bromo-2,4-dimethoxybenzene (9):

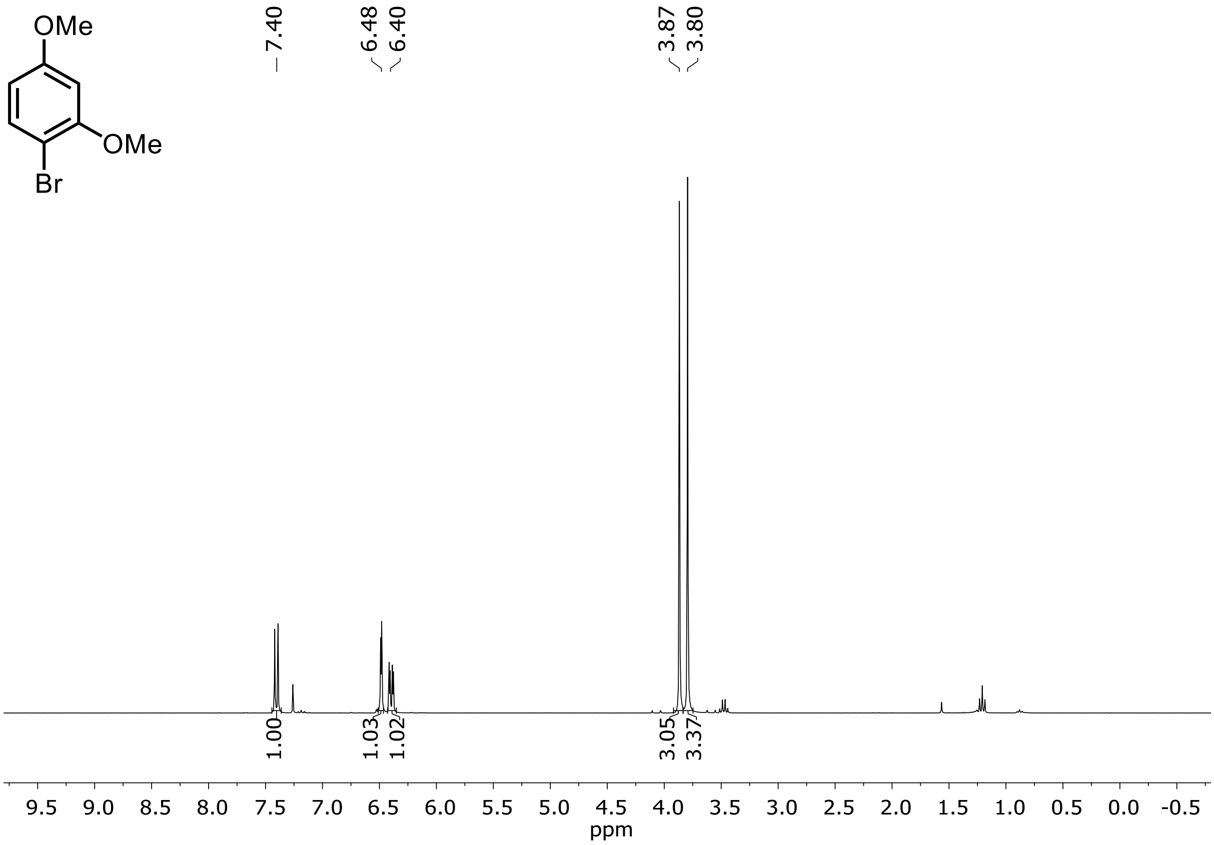

^{1}H NMR (300 MHz, $CDCl_3$) of compound **9**.

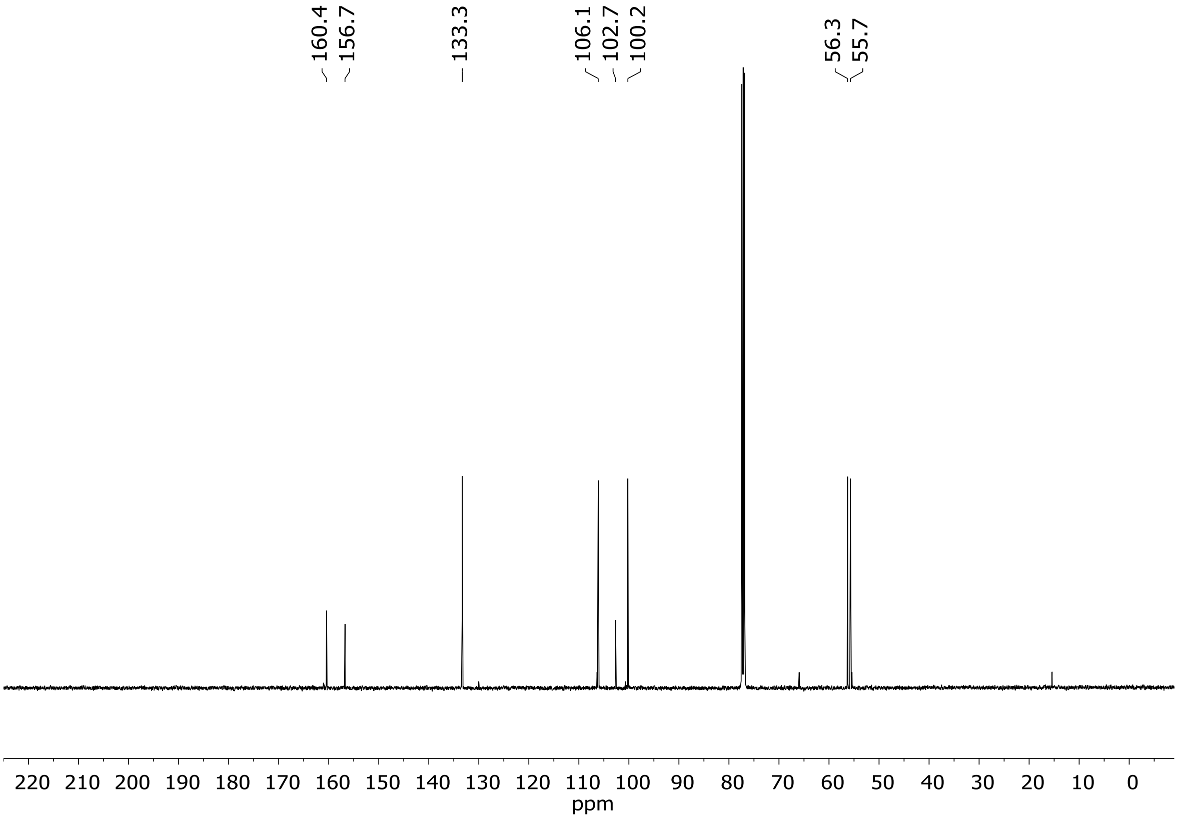

^{13}C NMR (125 MHz, $CDCl_3$) of compound **9**.

5. References

[1] G. R. Fulmer, A. J. M. Miller, N. H. Sherden, H. E. Gottlieb, A. Nudelman, B. M. Stoltz, J. E. Bercaw, K. I. Goldberg, *Organometallics* **2010**, *29*, 2176-2179.

[2] B. Sun, H. Tian, R. Ding, J. Li, W. Jiao, M. Han, Y. Liu, *Synthesis* **2018**, *50*, 4325-4335.

[3] S. Song, X. Li, X. Sun, Y. Yuan, N. Jiao, *Green Chem.* **2015**, *17*, 3285-3289.

[4] G. Y. Ishmuratov, R. Y. Kharisov, M. P. Yakovleva, R. R. Muslukhov, E. G. Galkin, V. S. Shmakov, T. V. Khakimova, G. A. Tolstikov, *Russ. Chem. Bull.* **2000**, *49*, 717-721.

[5] A. Terent'ev, I. Krylov, D. Borisov, G. Nikishin, *Synthesis* **2007**, 2979-2986.

[6] X. Xiong, F. Tan, Y. Y. Yeung, *Org. Lett.* **2017**, *19*, 4243-4246.

[7] K. D. Collins, F. Glorius, *Nat. Chem.* **2013**, *5*, 597-601.

[8] K. D. Collins, A. Ruhling, F. Glorius, *Nat. Protoc.* **2014**, *9*, 1348-1353.
